# A human stem cell-derived test system for agents modifying neuronal *N*-methyl-d-aspartate-type glutamate receptor Ca^2+^-signalling

**DOI:** 10.1007/s00204-021-03024-0

**Published:** 2021-03-13

**Authors:** Stefanie Klima, Markus Brüll, Anna-Sophie Spreng, Ilinca Suciu, Tjalda Falt, Jens C. Schwamborn, Tanja Waldmann, Christiaan Karreman, Marcel Leist

**Affiliations:** 1 In Vitro Toxicology and Biomedicine, Department Inaugurated by the Doerenkamp-Zbinden Foundation, University of Konstanz, Universitaetsstr. 10, 78467 Konstanz, Germany; 2 Cooperative Doctorate College InViTe, University of Konstanz, Konstanz, Germany; 3 Konstanz Research School Chemical Biology (KoRS-CB), University of Konstanz, Konstanz, Germany; 4 Developmental and Cellular Biology, Luxembourg Centre for Systems Biomedicine (LCSB), University of Luxembourg, 7, Avenue des Hauts-Fourneaux, 4362 Esch-sur-Alzette, Luxembourg; 5 CAAT-Europe, University of Konstanz, Konstanz, Germany

**Keywords:** Ketamine, MEA, Phencyclidine, Dextromethorphan, Domoic acid, Neurotoxicity, Ibotenic acid

## Abstract

Methods to assess neuronal receptor functions are needed in toxicology and for drug development. Human-based test systems that allow studies on glutamate signalling are still scarce. To address this issue, we developed and characterized pluripotent stem cell (PSC)-based neural cultures capable of forming a functional network. Starting from a stably proliferating neuroepithelial stem cell (NESC) population, we generate “mixed cortical cultures” (MCC) within 24 days. Characterization by immunocytochemistry, gene expression profiling and functional tests (multi-electrode arrays) showed that MCC contain various functional neurotransmitter receptors, and in particular, the *N*-methyl-d-aspartate subtype of ionotropic glutamate receptors (NMDA-R). As this important receptor is found neither on conventional neural cell lines nor on most stem cell-derived neurons, we focused here on the characterization of rapid glutamate-triggered Ca^2+^ signalling. Changes of the intracellular free calcium ion concentration ([Ca^2+^]_i_) were measured by fluorescent imaging as the main endpoint, and a method to evaluate and quantify signals in hundreds of cells at the same time was developed. We observed responses to glutamate in the low µM range. MCC responded to kainate and α-amino-3-hydroxy-5-methyl-4-isoxazolepropionic acid (AMPA), and a subpopulation of 50% had functional NMDA-R. The receptor was modulated by Mg^2+^, Zn^2+^ and Pb^2+^ in the expected ways, and various toxicologically relevant agonists (quinolinic acid, ibotenic acid, domoic acid) triggered [Ca^2+^]_i_ responses in MCC. Antagonists, such as phencyclidine, ketamine and dextromethorphan, were also readily identified. Thus, the MCC developed here may fill an important gap in the panel of test systems available to characterize the effects of chemicals on neurotransmitter receptors.

**Supplementary Information:**

The online version contains supplementary material available at 10.1007/s00204-021-03024-0.

## Introduction

Identifying compounds that interfere with ionotropic glutamate receptor signalling is important for the safety evaluation of drugs and environmental chemicals. Such information is of utmost toxicological relevance, as l-glutamate is the main excitatory neurotransmitter in the brain (Fukaya et al. 2003; Maycox et al. 1988). Therefore, modulation of glutamate signalling may lead to many adverse effects. The many receptors of this acidic amino acid can be divided into metabotropic receptors and ionotropic receptors. The latter group can be further subdivided into *N*-methyl-d-aspartate (NMDA) receptors and non-NMDA receptors (kainate and AMPA subtypes) (Traynelis et al. 2010).

The NMDA receptor (NMDA-R) plays a critical role in synaptic plasticity in the hippocampus, i.e. for memory and learning (Malenka and Nicoll 1993; Morris 2013). Disturbed signalling of the receptor is assumed to contribute to neurodegenerative diseases (Alzheimer’s disease, Huntington’s disease) (DiFiglia 1990; Liu et al. 2019), neurological disorders like epilepsy and stroke (EpiPM 2015; Lipton 1999), and neuropsychiatric disorders like schizophrenia (Cohen et al. 2015). Therefore, several agonists, antagonist and modulators of the NMDA-R have been developed as drug candidates or pharmacological tools: NMDA has been identified as a specific agonist for the NMDA-R; the NMDA-R antagonist ketamine is used clinically as an anaesthetic (Sinner and Graf 2008); the receptor blocker memantine is an approved medication for Alzheimer’s disease (Witt et al. 2004); and some NMDA-R antagonists, e.g., phencyclidine (PCP) and dextromethorphan (DXM) cause hallucinogenic and dissociative symptoms and are used as illicit recreational drugs (Williams and Lundahl 2019).

Biochemically, the NMDA-R is a heterotetramer consisting of two different subunit classes, namely the glycin-binding NR1 (gene: *GRIN1*) and the glutamate-binding NR2 (Ulbrich and Isacoff 2008). The latter has the different isoforms NR2A, NR2B, NR2C and NR2D (genes: *GRIN2A-D*) with distinct spatial and temporal expression patterns (Cull-Candy et al. 2001). Specifically, the subunit composition of the NMDA-R changes during the development of the CNS. Most changes occur prenatally. In human cortex and hippocampus, *GRIN2B* is expressed at relatively constant levels, while *GRIN2A* expression levels increase during brain development and early postnatal life (Bar-Shira et al. 2015). The heteromeric GRIN1/GRIN2B receptor is more prevalent amongst extrasynaptic NMDA-R than, e.g. the GRIN1/GRIN2A complex (Tovar and Westbrook 1999).

For receptor activation, sufficient occupancy of both the agonist (Glu) and the co-agonist sites (glycine or d-serine) needs to be reached (Johnson and Ascher 1987). In addition, the postsynaptic membrane has to be depolarized so that the physiological Mg^2+^ block is relieved (Cull-Candy et al. 2001). When the NMDA-R channel opens, Na^+^ and Ca^2+^ can enter the cell (Balu 2016). The influx of Ca^2+^ into the postsynaptic neuron triggers many specific signalling pathways (Papadia and Hardingham 2007). The activity of the receptor as an ion channel can be inhibited with general antagonists of the glutamate site [e.g. AP5 (Hansen et al. 2018)], antagonists of the glycine site [e.g. kynurenic acid (Zhou et al. 2012)], specific antagonists against NR2 isoforms [e.g. traxoprodil selective for NR2B (Chenard et al. 1995)] or universal channel blockers, [MK801, memantine, PCP, DXM, ketamine (Hansen et al. 2018)].

As the NMDA-R is involved in many diseases and toxicological AOPs (Chen et al. 2020; Liu et al. 2019; Sachana et al. 2019; Tschudi-Monnet and FitzGerald 2019; Wang and Reddy 2017), agents interfering with its function are a major health concern and need to be identified. An in vitro test method for this purpose would ideally employ human cells and measure a physiological change directly linked to ionotropic glutamate receptors. There is a range of possibilities to establish test methods for glutamate receptor interactions. Typical pharmacological binding assays, as used for advanced drug candidates are at one end of the spectrum (Berger et al. 2012; Pottel et al. 2020). The disadvantages of these assays are the uncoupling from the natural physiological environment, and that adversity is hard to define. Traditional animal studies are on the other end of the spectrum. Besides issues of species correlation and ethical aspects, these models have disadvantages concerning the exact target definition and the throughput. In between, there are many cellular and tissue-based models (Hondebrink et al. 2016; Meijer et al. 2019; Nehme et al. 2018; Yamazaki et al. 2016). Some of them allow both measurements of very early events (in the sense of initial key events of an AOP), but also more complex downstream disturbances, as proxy for an adverse outcome.

Modelling the human brain for toxicity studies is challenging, as test systems should include the various cell types the human brain is composed of. This may be achieved by the generation of mixed neuronal cultures from pluripotent stem cells (Heikkilä et al. 2009; Russo et al. 2018; Sasaki et al. 2019). Such a test system should include excitatory and inhibitory neurons, to be able to form self-regulating neuronal networks that also can be modulated. Another important cell type is astrocytes, as they are important modulators of neuronal signalling (Ishii et al. 2017; Tukker et al. 2018). The conditio sine qua non is that (i) at least some of the cells express ionotropic glutamate receptors, in particular NMDA-R, that (ii) they react to known pharmacological agonists, and (iii) that this reponse can be quantified.

One commonly used method to investigate neurotransmitter activity is to measure their effect on the free intracellular Ca^2+^ concentration ([Ca^2+^]_i_) (Leist and Nicotera 1998b; Nicotera et al. 1999). To this end, cells are stained with a Ca^2+^-sensitive dye to record its fluorescence changes (Karreman et al. 2020; Leist et al. 1997b; Miyawaki et al. 1997; Volbracht et al. 2006). Another method is to culture cells on microelectrode arrays (MEA). These arrays record the extracellular field potential of cultured neurons and thereby give a comprehensive overview of the electric activity of cultured neuronal networks (Hofrichter et al. 2017; Hogberg et al. 2011; Nimtz et al. 2020; Shafer 2019; Strickland et al. 2018; Vassallo et al. 2017).

In the past, it has been notoriously difficult to set up cell-based test systems for NMDA-R, as the activity of this receptor can lead to cell death by excitotoxicity (Leist and Nicotera 1998a; Nicotera et al. 1999). Assays used in the past maintained the cells in the presence of antagonists that were washed out before testing (Bettini et al. 2010; Feuerbach et al. 2010; Guo et al. 2017). Repeated testing or follow-up of downstream effects is not possible in such systems. We, therefore, developed a novel mixed neuronal culture, in which NMDA-R expressing cells can survive. We set out to characterize this test system and to establish Ca^2+^ imaging as a quantitative endpoint. Potential applications were demonstrated by assessment of agonists and by profiling of toxicologically relevant inhibitors.

## Materials and methods

### Materials

l-Glutamine, cAMP, apotransferrin, glucose, insulin, putrescine, selenium, progesterone, ascorbic acid, AP5, domoic acid, ketamine, quinolinic acid, traxoprodil, nicotine, dextromethorphan (DXM), MK801, and bicuculline were purchased from Sigma (St. Louis, USA). DMEM/F12, knockout serum replacement, Neurobasal, N2 supplement, B27 without ascorbic acid supplement, Glutamax, NEAA, ß-mercaptoethanol, HBSS, Triton-X-100, PBS, H-33342, FBS, Trizol, Fluo-4 Direct™ Calcium Assay Kit were purchased from ThermoFisher Scientific (Waltham, USA). GDNF, noggin, BDNF and NGF were purchased from R&D systems (Minneapolis, USA). Dorsomorphin, SB-431642, SU5402, ibotenic acid, NMDA, S-AMPA, phencyclidine (PCP), and kainate were purchased from Tocris (Bristol, UK). Chir99021 was purchased from Axon Medchem (Groningen, Netherlands); DAPT was purchased from Merck Millipore (Billerica, USA). Matrigel was purchased from Corning (Corning, USA); iScript and SsoFast™ EvaGreen® Supermix were purchased from BioRad (Hercules, USA). EdU click Kit was purchased from baseclick (Neuried, Germany). Purmorphamine (PMA) was purchased from Enzo (Farmingdale, USA). Veratridine was purchased from Alomone labs (Jerusalem, Israel). TGFß was purchased from Peprotech (Rocky Hill, USA).

### Differentiation and maintenance of NESC

The pluripotent stem cell line WA09 line (H9) (Balmer et al. 2014; Dreser et al. 2020; Thomson et al. 1998) was obtained from WiCell (Madison, WI, USA) and the line iPSC EPITHELIAL-1 (= Sigma 0028) was obtained from Sigma. The pluripotent stem cells were differentiated with a protocol adapted from Reinhardt et al. (2013) into neuroepithelial stem cells (NESCs) via embryoid body (EB) formation. To distinguish the differentiation protocols here, we used “days of differentiation prime” (DoD’) for the generation of NESC from PSC, and “days of differentiation” (DoD) for the protocol leading from NESC to MCC. On DoD0’ colonies of PSC were detached with Accutase and transferred to non-coated dishes to spontaneously form EBs in EB medium (KnockOut DMEM, 25% serum replacement, 2.5 mM l-glutamine, 1 × nonessential amino acids, and 100 µM µM ß-mercaptoethanol) supplemented with 10 µM SB-431542, 1 µM dorsomorphin, 3 µM CHIR99021, and 0.5 µM purmorphamine. On DoD2′, the medium was changed to differentiation medium (N2B27 medium: 50% Dulbecco’s modified Eagle’s medium/F12 [DMEM/F12], 50% neurobasal medium, 2 mM l-glutamine, 1 × B27 without vitamin A, and 1 × N2 (all purchased from Gibco, Carlsbad, USA)) supplemented with 10 µM SB-431542, 1 µM dorsomorphin, 3 µM CHIR99021, and 0.5 µM purmorphamine. On DoD4′ medium was changed to NESC maintenance medium (N2B27 medium supplemented with 150 µM ascorbic acid, 3 µM CHIR99021, and 0.5 µM purmorphamine). At DoD6′, EBs were disaggregated, transferred to Matrigel-coated six-well plates in a 1:6 dilution, and cultured for seven days with a medium change (NESC maintenance medium) every other day. After seven days, cells were detached with accutase and split in a 1:5 ratio. Cells were always split when reaching confluency of 75%. After three passages, NESCs were cryopreserved in NESC maintenance medium containing 10% DMSO without serum addition. This allowed the production of working stocks of the same NESCs. For maintaining a NESC population, cells were thawed and cultured in NESC maintenance medium with medium changes every other day. Cells were split when confluency of about 75% was reached. Cells were used for differentiation into MCCs between passage 4 and 20.

### Differentiation of MCCs from NESCs

A single-cell suspension of NESC was seeded at a density of 28,000 cells/cm^2^ on Matrigel-coated plates in NESC maintenance medium. After 2 days, defined here as day of differentiation 2 (DoD2) the medium was replaced by neuronal differentiation medium (N2B27 supplemented with 10 ng/ml BDNF, 10 ng/ml GDNF, 1 µM TGFß3, 500 µM cAMP, 200 µM ascorbic acid). Under these conditions, cells were left to differentiate into MCCs for > 20 days. On DoD4, DoD7, DoD10, half of the medium was changed. After this time, half of the medium was changed twice a week.

### Differentiation of peripheral neurons

The iPSCs were differentiated into immature dorsal root ganglia neurons according to Hoelting et al*.* (2016) with the following minor changes. Cells were seeded at a density of 90,000 cells/cm^2^ on Matrigel. The differentiation was started by addition of neural differentiation medium (KSR-S; Dulbecco’s modified Eagle’s medium [DMEM/F12] with 15% knockout serum replacement, 1 × Glutamax, 1 × nonessential amino acids, and 50 µM ß-mercaptoethanol) supplemented with 17.5 ng/ml noggin, 10 µM SB-431642 from DoD0 to DoD5. From DoD2 on, the three small molecules CHIR99021 (1.5 µM), SU5402 (5 µM) and DAPT (5 µM) were added. From DoD4′ onwards, the medium was gradually replaced by N2-S medium (DMEM/F12, with 2 mM Glutamax, 0.1 mg/ml apotransferrin, 1.55 mg/ml glucose, 25 mg/ml insulin, 100 mM putrescine, 30 nM selenium, and 20 nM progesterone). After differentiating for nine days, the cells were cryopreserved. The cells were thawed and subsequently seeded at a density of 100,000 cells/cm^2^ in 25% KSR-S and 75% N2-S supplemented with 1.5 µM CHIR99021, 5 µM SU5402 and 5 µM DAPT. On DoD1 and DoD2, 50% of the medium was changed. On DoD3 and DoD4 cells received N2-S medium, supplemented with 25 ng/ml BNDF, 25 ng/ml GNDF and 25 ng/ml NGF and 2 µM AraC. For further differentiation and maturation, half medium changes were performed every 3 days.

### Measurement of changes in intracellular Ca^2+^ concentrations

MCCs or peripheral neuron precursors were seeded in 96-well plates and differentiated as described above. Before measuring the changes in concentration of intracellular free Ca^2+^ ([Ca^2+^]_i_), differentiation medium was changed to artificial cerebrospinal fluid (aCSF): NaCl [140 mM], KCl [3 mM], CaCl_2_ [2.5 mM], MgCl_2_ [1 mM], Na_2_HPO_4_ [1.2 mM], pH 7.4, and the cells were loaded with Fluo-4 Direct™ Calcium Assay Kit and H-33342 for 30 min at 37 °C. For experiments requiring a pre-incubation phase, loading with Fluo-4 was performed in parallel. The changes in [Ca^2+^]_i_ were monitored with a VTI HCS microscope (ThermoFisher Scientific, Pittsburgh, USA) equipped with an incubation chamber providing an atmosphere with 5% CO_2_ at 37 °C. Test compounds were administered by an automated pipettor at 10 s after the first picture was taken. Images were taken as fast as possible for 45 s (approx. one image/second) and exported as .avi video files. The video files were afterwards analysed with the CaFFEE software (Karreman et al. 2020) to obtain single cell time-course information on [Ca^2+^]_i_. The cell densities and number of evaluated cells were very similar for all experimental conditions. However, the overall cell numbers shown in some figures may differ, because different numbers of experiments were included in the evaluation.

### Microelectrode array recordings

NESCs were seeded at a density of 25,000 cells/7 µl drop on Matrigel-coated 24-well CytoView MEA plates (16 electrodes per well). After 1 h, the well was filled up with 500 µl medium. The cells were then allowed to differentiate as described above. For time course experiments, the same plates were measured on different days. All plates were equilibrated for 10 min prior to recording (Maestro Edge, Axion Biosystems, Atlanta, USA), followed by 30 min of baseline recording. After the baseline recording, agonists/antagonists were added and recording was continued for another 30 min. All compounds were administered in 250 µl of neuronal differentiation medium. All recordings were captured using the Axion Integrated Studio Navigator (Axion Biosystems, Atlanta, USA) with a recording chamber at 37 °C and 5% CO_2_. For raw data acquisition, signals from all electrodes were recorded simultaneously with a sampling frequency of 12.5 kHz/channel. The recorded raw files were converted offline from voltage traces into various time-dependent data sets, such as spiking frequency. The threshold spike detector was set to 5.5 × of the noise level (signal SD) on each electrode, using adaptive threshold crossing for spike detection. Bursts were detected by setting inter-spike intervals (ISI) to ≤ 100 ms and requiring minimum 5 spikes per second. Network bursts were defined by the same ISI of ≤100 ms, a minimum of 50 spikes per second and at least 60% of active electrodes involved in bursting. To analyse the acute effects of agonists and antagonists, the number of spikes was binned (bin size = 0.1 s) and shown over time in comparison to baseline.

### Immunofluorescence staining and microscopy

Cells were grown on Matrigel-coated coverslips and fixed with 4% paraformaldehyde. Then they were permeabilised in 0.3% Triton X-100 and blocked for 1 h in PBS containing 5% fetal bovine serum and 0.1% Triton X-100. Primary antibodies (see Table S2) were administered and kept for 1 h at room temperature, followed by washing and incubation with secondary antibodies and Hoechst H-33342 for 30 min. For each condition, five images of three biological replicates were taken with a Zeiss Axio Observer with ZEN 2 pro blue edition software or a Zeiss LSM 880 and further processed with ImageJ (Version 1.52p). On each picture between 45 and 60 cells were captured, following this between 650 and 900 cells were evaluated and a representative picture is shown.

### RNA extraction, cDNA synthesis and real-time qPCR

The extraction of total RNA was performed with TRIzol, according to the manufacturer's protocol, followed by the reverse transcription of 1 µg total RNA with iScript. cDNA was quantified using the SsoFast™ EvaGreen® Supermix. The threshold cycle (*C*_T_) was determined for each sample, using the CFX data analysis software (Bio-Rad, USA). Reference genes were used and mRNA levels of different genes of interest were compared to them at different time points of the differentiation using the ΔΔ method (Livak and Schmittgen 2001). For a detailed list of primers used in this study, see Table S3.

### Transcriptome data generation and analysis

Sample preparation was performed by removing the medium from each well and lysing the cells immediately in 25 µl of 1 × Biospyder lysis buffer (BioSpyder Tech., Glasgow, UK). Samples were stored at – 80 °C until shipment to Bioclavis (BioSpyder Tech., Glasgow, UK) on dry ice. Transcriptomics data were determined by TempO-Seq technology (BioSpyder Tech., Glasgow, UK), a targeted RNA-sequencing method developed by BioSpyder Technologies, Inc. (House et al. 2017). The set of genes analysed, and the read data are detailed in Supplement file 2, organized as Excel workbook, including labelling and clear explanations.

For the data analysis, the R package DESeq2 (v1.24.0) was employed (Love et al. 2014). The DESeq2 object was constructed from raw counts of mRNA species and its size factors were normalized to total sample counts per million (CPM). A Wald test was used for the statistical analysis of differential gene expression in the treatment group (against the DMSO control group). For a gene to be considered significantly deregulated, the threshold of Benjamini–Hochberg adjusted *p* values was set to ≤ 0.05. A cutoff for fold changes (FC) was not introduced. Over-representation analysis of gene ontology terms (GO) was based on Fisher’s *F* test as implemented in g:profiler software (Raudvere et al. 2019).

### Statistics

Experiments were performed at least on three cell preparations, with several technical replicates for each cell batch. Descriptive statistics, transparent display of the data structure and experimental variability were included in display figures and supplementary information. For significance testing, GraphPad Prism 5 software (Version 7.04, Graphpad Software, Inc, San Diego, USA) was used. Data were evaluated by Fisher’s exact test or by *t* test (when two groups were compared). *P* values < 0.05 were regarded as statistically significant.

## Results

### Generation and phenotyping of mixed cortical cultures (MCCs)

To generate mixed cortical cultures (MCCs) expressing neurotransmitter receptors, we used a two-step protocol. First, pluripotent stem cells (pSCs) were differentiated into a proliferating neuroepithelial stem cell population (NESC) (Fig. S1A) (Reinhardt et al. 2013). These cells did not express the pluripotency markers NANOG and OCT4 and could be cultivated for up to 20 passages (Fig. S1B). During this time, the expression of typical NESC markers HES5, PAX6, and DCX was stable (Fig. S1C), while PAX3 and NES even increased expression levels (Fig. S1D). The activity of cell cycle genes (CDK1, CDK2, CCND1, and CCND2) remained constant (Fig. S1E). Immunostaining confirmed the presence of Sox1, Pax3, Pax6, doublecortin, and nestin protein, as well as of the proliferation marker Ki67 (Fig S1F).

**Fig. 1:**
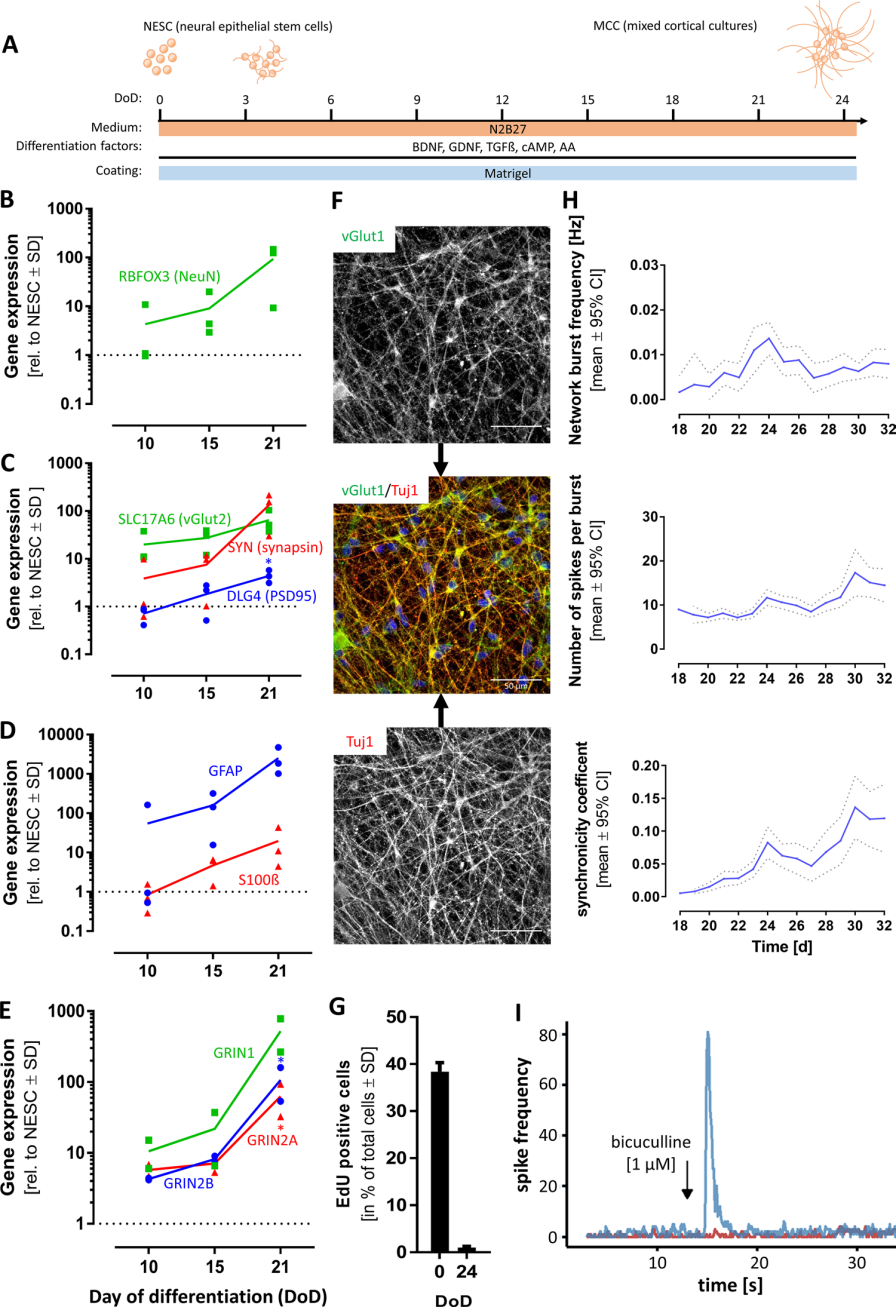
Characterization of mixed cortical cultures (MCCs). Mixed cortical cultures (MCCs) were generated from NESCs according to Reinhardt et al. (2013), by differentiating them for > 20 days on Matrigel in neuronal differentiation medium. **a** Schematic display of MCC differentiation (*BDNF* brain-derived neurotrophic factor, *GDNF* glial cell-derived neurotrophic factor, *TGFß* transforming growth factor beta, *cAMP* cyclic adenosine monophosphate, *AA* ascorbic acid). **b** Gene expression profile of the neuronal marker RBFOX3. Gene expression was quantified by real-time PCR. Data are given relative to reference genes (RPL13A and TBP) and the NESC starting population (= DoD0, depicted as dotted line) for DoD10, DoD15, and DoD21. **c** Gene expression of synaptic markers. Gene expression of **d** glial markers and of **e** NMDA receptor sub-units NR1 (GRIN1), NR2A (GRIN2A) and NR2B (GRIN2B). The symbols show the values of single biological replicates and the coloured lines show their mean. Significance was tested with a *t* test; *p value < 0.05. **f** Immunofluorescence image of MCCs on DoD24 using antibodies against the neuron-specific cytoskeletal marker beta-III-tubulin (Tuj1) and the vesicular glutamate transporter 1 (vGlut1). The composite images also include the nuclei stained with H33342 (blue) Scale bar: 50 µm. **g** Cells on DoD0 and DoD24 were allowed to incorperate the nucleotide analog EdU, to visualize mitotic activity. EdU-positive cells were counted and are shown as percentage of total cell number ± SD. **h** MCCs were differentiated on MEA plates. Spontaneous spikes of electrical activity were recorded on various days of differentiation for the same cells. From these activity measurements (30 min each day), the parameters “network burst frequency”, “number of spikes per burst”, and “synchronizity coefficient” were calculated and plotted over time. The dotted line indicates the confidence interval. **i** On DoD24 MCCs on MEAs were treated with the GABA_A_ receptor antagonist bicuculline [1 µM] (blue, addition is indicated by the black arrow, baseline in red). The generation of spikes was recorded directly before and after administration, the number of spikes was binned (bin size 0.1 s) and a representative example of the acute response is shown. A full set of data over an extended time span is presented in Fig. S2 (color figure online)

As a next step, NESCs were differentiated for > 20 days by the addition of the five differentiation factors: BDNF, GDNF, cAMP, TGFß3 and ascorbic acid (Fig. 1a). During the time course of differentiation, the cells acquired the following features of mature neurons: (i) they formed a neuronal network with a complex and extensive neurite structure (Fig S2A); (ii) the typical marker of post-mitotic neurons, RBFOX3 (Fig. 1b), and synaptic markers, like SLC17A6, SYN, DLG4 (Fig. 1c) were upregulated. This was paralleled by the protein expression of NeuN (the product of the RBFOX3 gene), Map2, ßIII-tubulin, and synaptophysin (Fig S2B,C). The MCCs also included glial cells, as indicated by the gene expression of GFAP and S100ß (Fig. 1d) and immunopositivity for S100ß (Fig S2D); (iii) subunits of the NMDA-R (GRIN1, GRIN2A, and GRIN2B) increased their expression levels over differentiation time (Fig. 1e). The vesicular glutamate transporter 1 (vGlut1), a marker of glutamatergic neurons (Ito et al. 2015), was strongly expressed in MCCs (Fig. 1f); (iv) MCCs contained only 1% of dividing cells (Fig. 1g), while 40% of NESC incorporated EdU within the same labelling period. (Fig S2D). Thus, the cells were mostly postmitotic, which is a defining feature of neurons.

The most important functional features of mature neurons are their individual electrical activity and the formation of functional networks (Hogberg et al. 2011; Strickland et al. 2018). To examine these two features, MCCs were cultured on MEA, and their spontaneous electrical activity was recorded on different DoDs. Network bursts were observed from about DoD18 on. The frequency increased until DoD24 and then remained stable until DoD32. The number of spikes included in a burst increased slightly from DoD18 until DoD32. The same was observed for the synchronicity coefficient, a measure of coordination between different areas of the network (Fig. 1h). These data show that MCCs were electrically active and that their network properties required at least three weeks to develop. To test whether the spontaneous network activity could be modified by pharmacological intervention, DoD24 MCCs were treated with the GABA_A_ antagonist bicuculline (BIC). The application of 1 µM BIC resulted in a massive increase in spike frequency as an acute response (Fig. 1i). This period of pronounced electrical activity was followed by a short period of anergy (approx. 550 s) and then established a new steady state that showed higher activity than the untreated control cultures (Fig. 1e). All these features show that MCCs represent a mature neuronal culture, comprising various types of neurons (e.g. glutamatergic and GABAergic).

### Gene expression profiling of MCCs

**Fig. 2:**
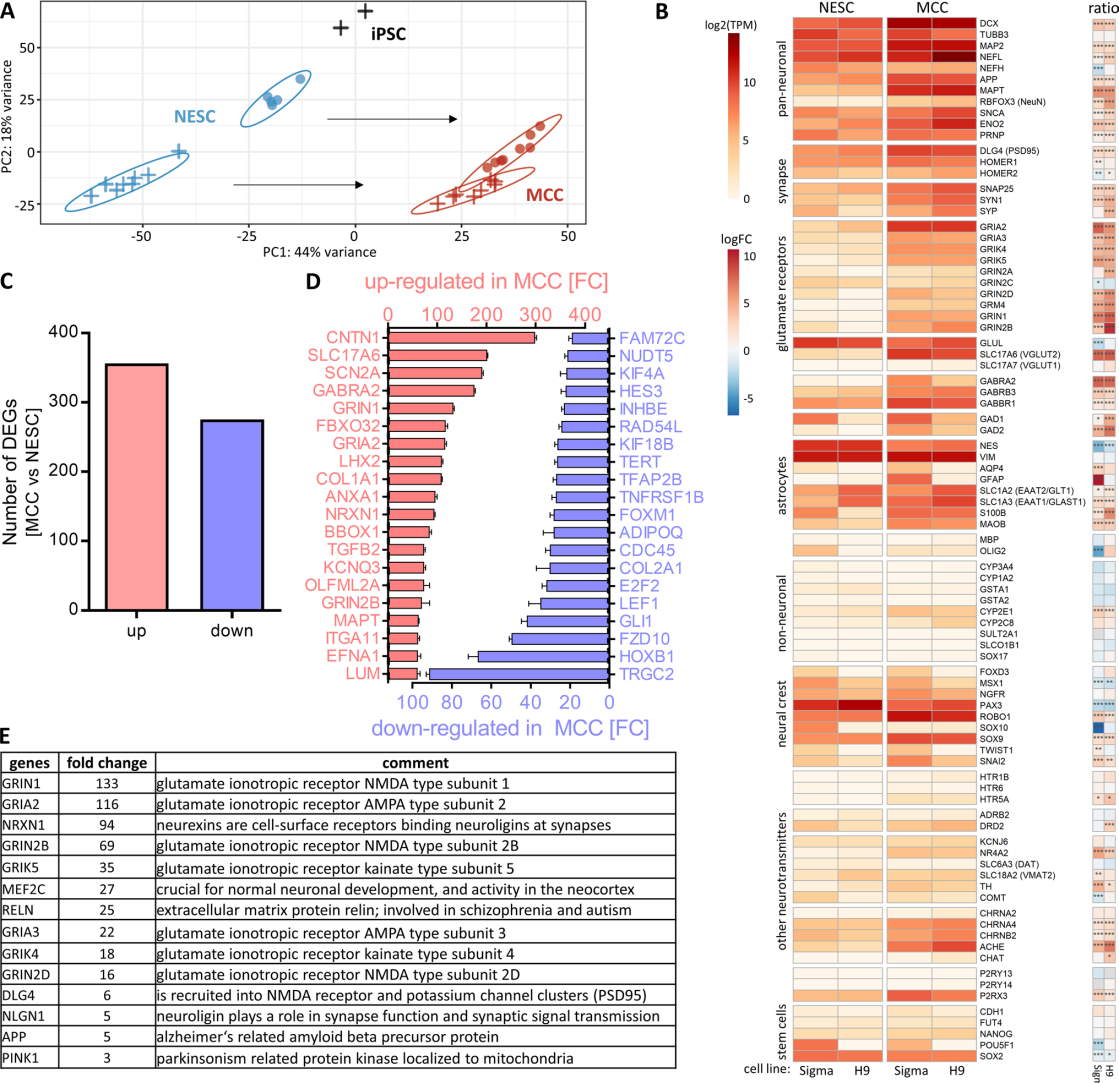
Transcriptome analysis of NESC and MCCs. Neuroepithelial stem cells (NESCs) from two pluripotent stem cell lines (H9 and Sigma iPSC) were differentiated into mixed cortical cultures (MCCs), and mRNA was prepared for transcriptome analysis. A set of about 3500 genes was used for targeted RNAseq analysis (TempOSeq method), and expression levels were obtained for four independent cell preparations (some with two samples per cell preparation). **a** A PCA was obtained for the three developmental stages, iPSC (*n* = 2), NESCs (*n* = 4), and MCCs (*n* = 4). The H9-derived cells are indicated by a filled circle. A cross indicates Sigma hiPSC-derived cells. The axes are scaled according to the variance covered and the black arrows indicate the main differentiation effect in the two-dimensional PCA space. **b** The heatmap shows the expression of selected neuronal, non-neuronal and stem cell markers for two cell lines (H9 and Sigma iPSC). Data to the left are given as absolute expression values in transcripts per kilobase million (TPM). In the small heatmap to the right data are given on a log_2_ fold change (FC) scale with MCC relative to NESC where red colours indicate increased expression in MCC. *p_adj_ < 0.05, **p_adj_ < 0.01, ***p_adj_ < 0.001. **c** Number of differentially expressed genes (DEGs) from the overlap of H9-derived cells and iPSC-derived cells is shown for upregulations (red) and downregulations (blue) with an adjusted *p *value ≤ 0.05. **d** Out of the DEGs in C, the top 20 up-regulated and down-regulated genes according to their fold change (FC) are shown. The error bars represent the standard deviation. **e** All upregulated DEGs with an adjusted *p *value ≤ 0.05 and log2FC > 1 were analysed for over-represented gene ontology (GO) terms (Raudvere et al. 2019). GO terms with a size between 4 and 1000 genes were included in further analysis. The first (lowest *p* value) 50 GOs of the category biological process were further assigned to the four key biological processes “neurodevelopment”, “synapse”, “neurotransmitter”, and “neuronal subtype (glutamate)”. The 14 genes relating to the glutamate system and being involved in the other three key biological processes are listed with their respective fold change and a short description for each gene. More data are displayed in Fig. S3

Throughout this study, two cell lines were used. They behaved phenotypically in a similar way. Transcriptome data were used to further explore their similarity. A first overview of the data structure was obtained by a principal component analysis (PCA) (Fig. 2a). NESC and MCC differed clearly from pluripotent source cells. The differentiation process moved NESC of both lineages in a similar way along the first principal component axis (PC1). NESC of the two cell lines showed some differences, but the final MCC populations were overlapping in their profiles. This analysis also showed that the differentiation was highly reproducible, as 16 samples from two cell lines yielded highly similar MCC populations.

To get a closer insight into neuronal characteristics of MCCs, absolute expression values for genes related to neuronal lineages, neurotransmitters, and non-neuronal lineages were compared. This analysis showed that pan-neuronal markers (like NEFL, MAPT, and MAP2) had a higher expression in MCCs than in NESC. Genes related to glutamate receptors (like GRIA, GRIK, GRIN and SLC17A6/7) were hardly expressed in NESC but were highly expressed in MCCs. Also, genes coding for GABA receptors (GABR) or synaptic markers were higher expressed in MCCs than in NESC. Furthermore, some genes related to the astrocyte lineage (SLC1A2/3, S100ß, MAOB) were upregulated. By contrast, the expression of neural crest genes and stem cell genes remained low and unchanged. Importantly, genes coding for proteins from other tissues, like liver (CYPs), were not expressed in MCCs (Fig. 2b, Table S4).

To allow a first overview of relative regulations, the ratios of NESC vs MCC expression were calculated confirming the general picture evident from an overview of the absolute levels. These regulation data provided additional evidence of the robustness of the differentiation protocol and revealed some minor differences between the cell lines. For instance, Sigma 0028 derived cells seemed to produce more mature astrocytes (AQP4, GFAP expression). This finding was, however, not followed up here, as the focus of our study was on neuronal (excitatory) signalling. In summary, this gene expression pattern was well in line with a mixed culture of neurons, with machinery in place for excitatory (glutamate) and inhibitory (GABA) neurotransmission. To investigate the similarity of the two cell lines used here (Sigma 0028 and H9) in more detail, their differentially expressed genes (DEGs) were plotted against each other. This scatter plot showed a high correlation (Fig. S3A), and a clustering analysis of the various cell populations confirmed that MCCs from both lineages clustered together, while they separated well from their precursors (Fig. S3B). As the variation between the two lines was not larger than variations between individual preparations of one line, we did not distinguish cell lines in the further course of our study.

Besides looking at preselected neural and non-neural genes, we also took an unbiased approach to gene expression analysis: the overall number of DEGs from the 3257 analysed genes were determined (about 600 DEGs) (Fig. 2c), and the genes that were most upregulated or downregulated (on a fold change basis) were compiled (Fig. 2d). Among the 20 most upregulated DEGs were the neurotransmitter receptors and channels SLC17A6, SCN2A, GABRA2, GRIN1, GRIA2, and GRIN2B. The most upregulated (about 300-fold) gene was CNTN1, a glycoprotein specific for neurons. Among the 20 most downregulated DEGs were genes involved in the Wnt/beta-catenin pathway (like FZD10), as well as GLI1, TERT, LEF1, HES3 which are involved in stem cell/precursor cell biology. Also HOXB1, a regulator for neural crest cells was strongly downregulated when NESCs were differentiated to MCCs. Genes generally involved in the cell cycle (E2F2, INHBE, FOXM1) were also amongst the most downregulated (Fig. 2d).

We also performed a gene ontology overrepresentation analysis. This resulted in over 400 overrepresented gene ontologies (oGOs) amongst upregulated DEGs. We assigned the 50 oGOs with lowest *p *values to the four key biological processes (KBPs) “neurodevelopment”, “neuronal subtypes (glutamate)”, “synapse”, and “neurotransmitter” according to Waldmann et al*.* (2014) (Fig. S3C). Following our interest in a test system that may be used to assess glutamate signalling, we looked for the genes common to the four KBPs. This resulted in a set of 14 genes (Fig S3D). Most of them were subunits of glutamate receptors or were involved in synapse function or formation (Fig. 2e). Five genes were in the KBP “neuronal subtypes (glutamate)” but not the other KBPs (Fig. S3E). For completeness, we also examined the 20 oGOs with the lowest *p* value for downregulation. They all dealt with regulation of cell cycle (Supplement 2), which confirms the conversion of a proliferating precursor population (NESC) to (postmitotic) neurons (MCC).

### Establishment of Ca^2+^ measurements as test endpoint

**Fig. 3:**
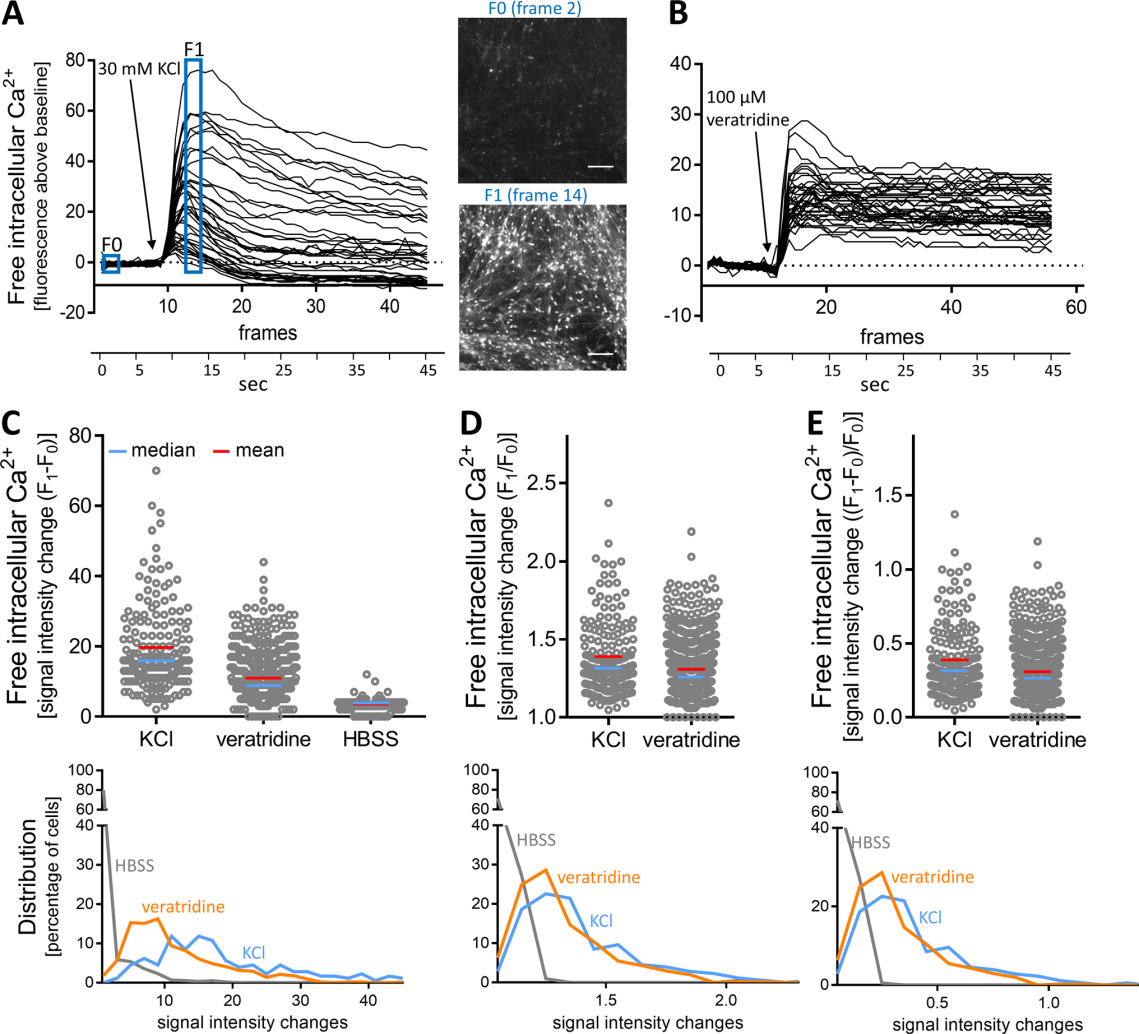
Quantification of Ca^2+^ imaging signals of control stimuli in MCCs. MCCs were used after a 24-day differentiation period for Ca^2+^ imaging. **a** Cells were depolarized with KCl [30 mM] and the fluorescence of the intracellular Ca^2+^ indicator was recorded over time. Raw data were processed by an imaging software that automatically identified cell bodies and produced fluorescence-time traces for every cell of the image field. These time traces were then aligned for baseline fluorescence (non-stimulated state), which was set to “zero”. This way, traces of absolute fluorescence changes (Δ signal intensity traces of 40 randomly selected cells are shown) due to the stimulus are displayed. To give an impression of the non-normalized data structure, fluorescence images at time points F_0_ and *F*_1_ are shown. Scale bar: 100 µm. **b** MCCs were treated with veratridine [100 µM] and data were processed as in (**a**). **c**–**e** To quantify the data, Δ signal intensity values were calculated from the baseline (*F*_0_) and peak (*F*_1_) data with three different methods: **c** *F*_1_−*F*_0_, **d** *F*_1_/*F*_0_, and **e** (*F*_1_−*F*_0_)/*F*_0_. The upper row shows the data distribution on a “per cell” basis. The lower row shows how signal intensity changes (bins of 3 fluorescence units) were distributed over the cell population

The hallmark of mature neurons is their communication by neurotransmitters. As excitatory neurotransmission is associated with a change in the intracellular free Ca^2+^ concentration ([Ca^2+^]_i_) (Leist and Nicotera 1998b), this process can be investigated by Ca^2+^ imaging (Tsien and Tsien 1990). Therefore, we set out to establish Ca^2+^ imaging as endpoint in the MCC test system. Initially, we used standard approaches to depolarize cells, such as an increase of K^+^ ions in the medium, or the opening of Na^+^ channels by the alkaloid veratridine (Hoelting et al. 2016; Scholz et al. 2011). The application of KCl [30 mM] (Fig. 3a) or veratridine [100 µM] (Fig. 3b) triggered a fast and strong increase in [Ca^2+^]_i_ in all individual cells. As data, obtained from a large number of cells over time, require further processing to allow quantification, we took two steps in this direction. First, a dedicated software (Karreman et al. 2020) identified the peak time point. Then fluorescence data were obtained for the ground state (*F*_0_) of each cell and for the peak time point (*F*_1_). Second, we compared the different approaches of the scientific literature (*F*_1_−*F*_0_; *F*_1_/*F*_0_; *F*_1_/*F*_0_−1) to calculate the fluorescence offset (Fig. 3c–e). A simple subtraction of F_0_ from *F*_1_ (Δ*F* = *F*_1_–*F*_0_) was found to give the best separation of stimulated cells (KCl, veratridine) and cells exposed only to buffer (HBSS) (Fig. 3c–e). This way, Ca^2+^ imaging allowed capturing signal changes in the complex MCC cultures.

To determine for an individual cell, whether it reacted to a stimulus, or not, we took a statistical approach to define an activation threshold. For this purpose, the Δ signal intensity values of > 2600 cells treated with HBSS were collected, and the data distribution, including average and the standard deviation of this data set, was determined (Fig. S4A). A range of three standard deviations was defined as the noise band on top of the data means. Accordingly, the upper noise level was a Δ*F* = 9 (Fig. S4B). Every cell with a Δ signal intensity value ≥ 9 was defined as a reactive cell. Following this approach, our endpoint had a false positive rate of 3.6% (Fig. S4C). When typical traces of cells treated with HBSS were followed over time (instead of peak time measurements), they did not cross the threshold of 9 at any time point (Fig. S4D). Based on the above procedure and findings, we used the signal threshold of 9 for further experiments to define reactive cells.

### Quantification of excitatory neurotransmitter response in MCCs

**Fig. 4:**
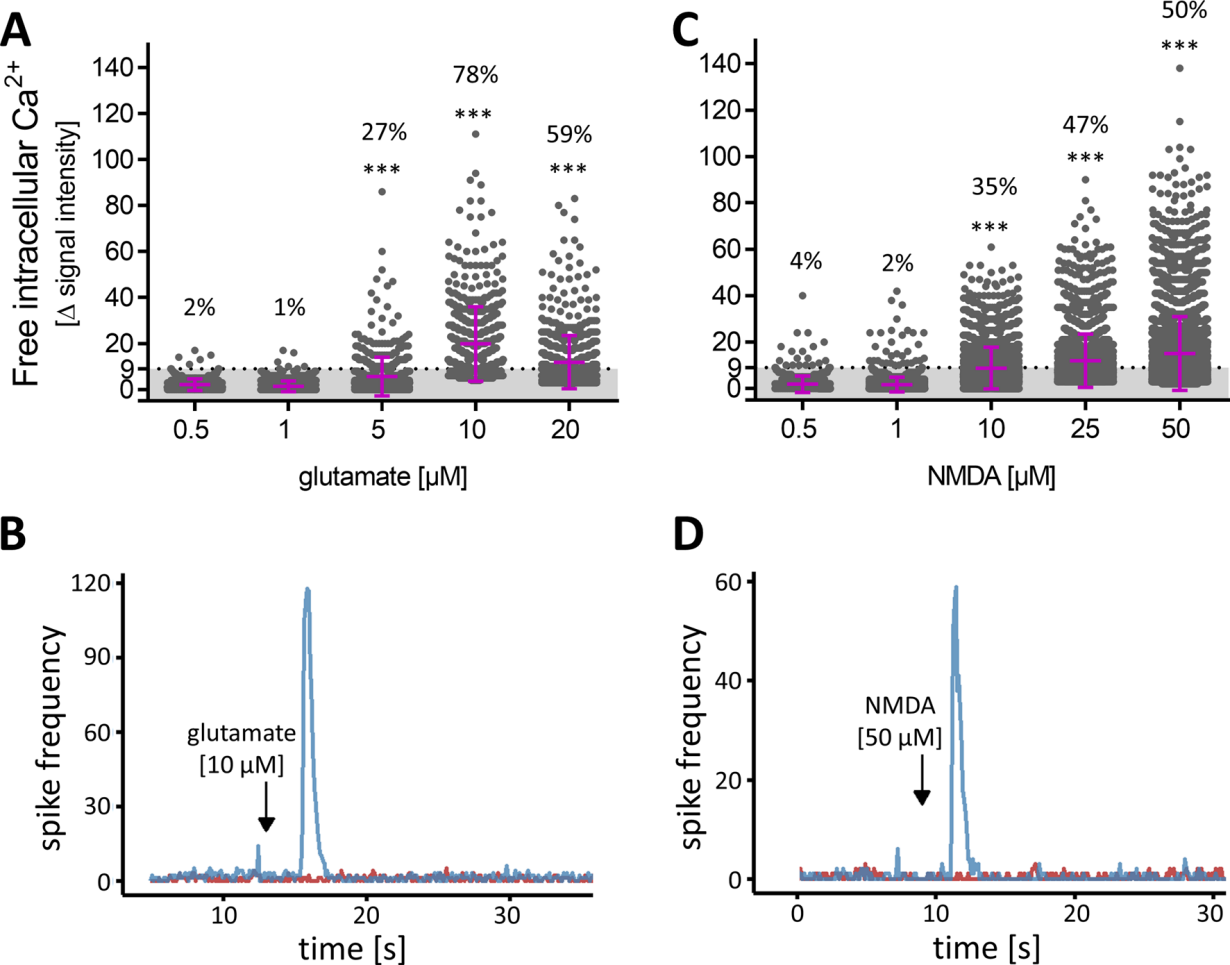
Concentration-dependent response of MCCs towards glutamate and NMDA. MCCs were differentiated for 24 days and subsequently used for Ca^2+^ imaging. **a** The changes of Ca^2+^ indicator fluorescence (= delta signal intensity) of each individual cell treated with glutamate is shown as a dot. The mean ± SD is shown in purple. The threshold of Δ signal intensity (≥ 9) is shown as dotted line the number above each bar indicates the percentage of cells with a Δ signal intensity above the threshold (= defined as reactive cell). Significance was tested with Chi-square test followed by exact Fisher’s test; ***p value < 0.001 relative to the lowest concentration. **b** On DoD24 MCCs on MEAs were treated with glutamate [10 µM] (blue, addition is indicated by the black arrow; baseline in red). The generation of spikes was recorded directly before and after administration and the number of spikes was binned (bin size 0.1 s). A respective example of the acute response is shown. **c** The changes of Ca^2+^ indicator fluorescence (= delta signal intensity) of each individual cell treated with NMDA is shown as a circle. The mean ± SD is shown in purple. The threshold of Δ signal intensity (≥ 9) is shown as dotted line and the number above each bar indicates the percentage of reactive cells. Significance was tested with Chi-square test followed by exact Fisher’s test; ***p value < 0.001 relative to the lowest concentration. **d** On DoD24 MCCs on MEAs were treated with NMDA [50 µM] (blue, addition is indicated by the black arrow; baseline in red). The generation of spikes was recorded directly before and after administration and the number of spikes was binned (bin size 0.1 s). A respective example of the acute response is shown (color figure online)

MCCs were exposed to low concentrations of glutamate on DoD24, and ≥ 5 µM glutamate led to a significant increase in the amount of reacting cells. At a concentration of 10 µM glutamate, 78% of cells were reactive (Fig. 4a). To confirm the specific response to glutamate by another well-established method, we measured the spike frequency on MEA after exposure of MCCs on DoD24 to glutamate [10 µM]. A clearly positive response was observed (Fig. 4b). For a selective stimulation of NMDA-R, we administered increasing concentrations of NMDA to MCCs. This resulted in a significant number of reactive cells at concentrations of ≥ 10 µM. At 50 µM NMDA, half of all cells were reactive (Fig. 4c). Moreover, the addition of NMDA [50 µM] was followed by a strong increase of the spike frequency on MEA (Fig. 4d). From the above data, we conclude that approx. 80% of MCCs express functional ionotropic glutamate receptors, and about 50% of the cells had a sufficient NMDA receptor expression to allow [Ca^2+^]_i_ responses. We pre-incubated MCCs with 10 µM glycine or 10 µM d-serine followed by glutamate [10 µM] or NMDA [50 µM] administration. The percentage of reactive cells did not change when cells were pre-incubated with the co-agonists (data not shown). Thus, exogenously added glycine or d-serine had no influence on the above-described effects of glutamate and NMDA.

In the next set of experiments, we selected potential negative controls to ensure the specificity of the NMDA response in MCCs. For this purpose, a strong activator of non-glutamate ionotropic receptors was used. We chose nicotine, as a nAChR ligand. Concentrations up to 50 µM (50 × the known EC_50_) did not trigger a [Ca^2+^]_i_ response (Fig. S5A). In another approach, we used human peripheral neurons. The positive control KCl [10 mM] triggered a pronounced increase in [Ca^2+^]_i_. No response was observed after the administration of NMDA [50 µM] (Fig. S5B). Taken together, these data show that MCCs contain a population of neurons that express functional NMDA-R, and react to an NMDA stimulus, but not e.g. to nicotine. A strong immediate reaction to NMDA is not observed in many stem cell-derived cultures, as also shown here for otherwise well-developed peripheral neurons.

### Investigation of ionotropic glutamate receptors in MCC cultures

**Fig. 5:**
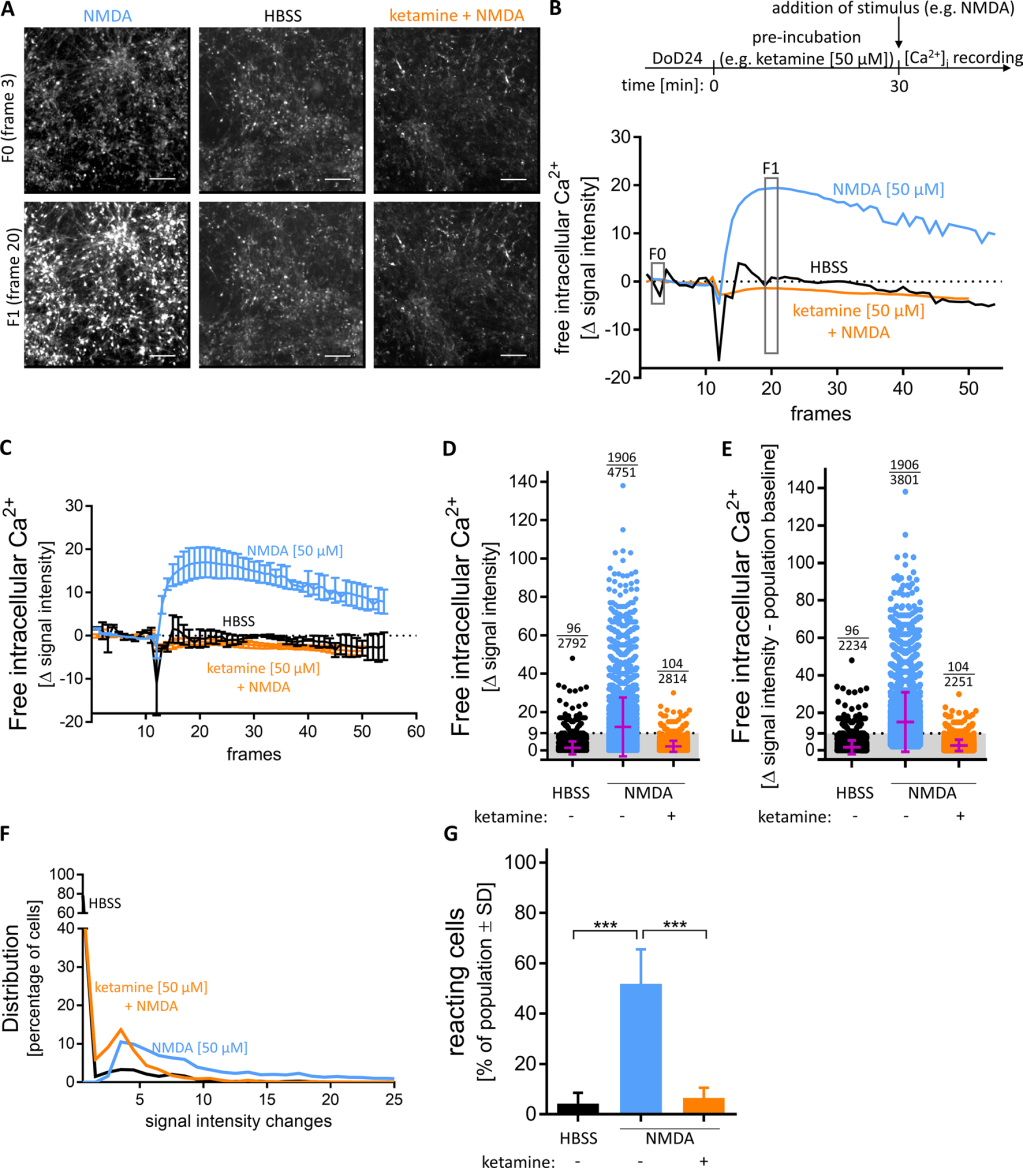
Different possibilities of analysis and depiction of Δ signal intensities. MCCs were differentiated for 24 days and subsequently used for Ca^2+^ imaging. **a** Images from cells treated with negative control HBSS, NMDA [50 µM], pre-incubated with ketamine [50 µM] for 30 min before addition of NMDA [50 µM], before stimuli addition (*F*0 (frame 3)), or after (*F*1 (frame 20)) are shown. Scale bar: 100 µm. **b** A schematic overview of Ca^2+^ imaging. Traces from wells shown in A. **c** Averaged traces of three individual wells treated with negative control HBSS or NMDA [50 µM], pre-incubated or not with ketamine [50 µM]. Data are means ± SD; *n* = 3. **d** The changes of Ca^2+^ indicator fluorescence (= delta signal intensity) of each individual cell treated with HBSS or NMDA [50 µM], pretreated or not with ketamine [50 µM], is shown as a circle. The mean ± SD is shown in purple. The threshold of Δ signal intensity (≥ 9) is shown as dotted line. The number above each column represents the number of reactive cells and the number of total cells analysed. **e** The Δ signal intensity of the population baseline was subtracted from the Δ signal intensity of each individual cell treated with HBSS or NMDA [50 µM], pretreated or not with ketamine [50 µM], and is shown as a circle. The mean ± SD is shown in purple. The threshold of Δ signal intensity (≥ 9) is shown as dotted line. The number above each column represents the number of reactive cells and the number of total cells analysed. **f** The data distribution of E is shown. **g** The percentage of cells with a Δ signal intensity ≥ 9 of the whole population was calculated for HBSS, NMDA [50 µM] pretreated or not with ketamine [50 µM]. Data are means ± SD; *n* = 3; ***p value < 0.001 (color figure online)

For further investigation of the functionality of the NMDA-R in MCCs, we used different receptor agonists and antagonists that have been often used in pharmacology and toxicology for system characterization. For instance, ketamine binds to the dizocilpine site of the NMDA-R (Sinner and Graf 2008) and thereby inhibits the ion influx of Na^+^ and Ca^2+^ into the post-synaptic neuron in a voltage-dependent manner (MacDonald et al. 1987). Ketamine is also used as a tool compound in schizophrenia research, as it models several disease symptoms. As NMDA-R dysfunction is considered an important basis of schizophrenia (Balu 2016), a system showing clear effects of such drugs can benefit this research. MCCs pre-incubated with ketamine [50 µM] had a completely blunted [Ca^2+^]_i_ response after NMDA [50 µM] application (Fig. 5a, b). To further control these data, we performed quantifications over the entire time course of several differentiations, which fully confirmed our findings for the peak time. For cells reactive to NMDA [50 µM] there was a fast increase in Δ signal intensity and a slower decline. In the presence of ketamine, we did not observe a signal, also after a considerable delay (Fig. 5c). The defined pharmacological responses to NMDA with/without its antagonist (ketamine) were used to confirm and refine our quantification approach for pharmaco-toxicological studies: single-cell fluorescence quantification showed that not all cells reacted to the same extent. MCCs treated with NMDA [50 µM] showed a widespread of Δ signal intensity, and about half of the cells remained below the activation threshold (Fig. 5d). MCCs most likely contain cells (e.g. glia or immature neurons) that do not react to NMDA. The quantification should ideally focus on the subpopulation of neurons with functional glutamate receptors. Therefore, we defined here the pool of absolutely non-reactive cells to be 20%, as this percentage did not react to glutamate [10 µM] (Fig. 4a). It was considered unlikely that cells would react to NMDA, but not to glutamate. Based on this assumption, the non-reactive population baseline (corresponding to 20% of all cells in a field) was removed from the analysis. This correction step had no visible effect on the apparent distribution and average signal intensity of cells treated with HBSS. For cells exposed to NMDA [50 µM], the average signal slightly increased and the lower boundary of the signal intensities were slightly higher. Cells pre-incubated with ketamine [50 µM] prior to NMDA [50 µM] behaved similar to those exposed to solvent (HBSS) (Fig. 5e). The frequency distribution of reactive cells showed that only after exposure to NMDA, there were Ca^2+^ signals above the threshold. For HBSS and ketamine pre-incubation, more than 95% of cells had a signal intensity change value below the baseline (≤ 9) (Fig. 5f). When the baseline-substracted data set was used to calculate the percentage of reacting cells, an excellent signal separation (NMDA vs NMDA + ketamine) was obtained (Fig. 5g).

**Fig. 6:**
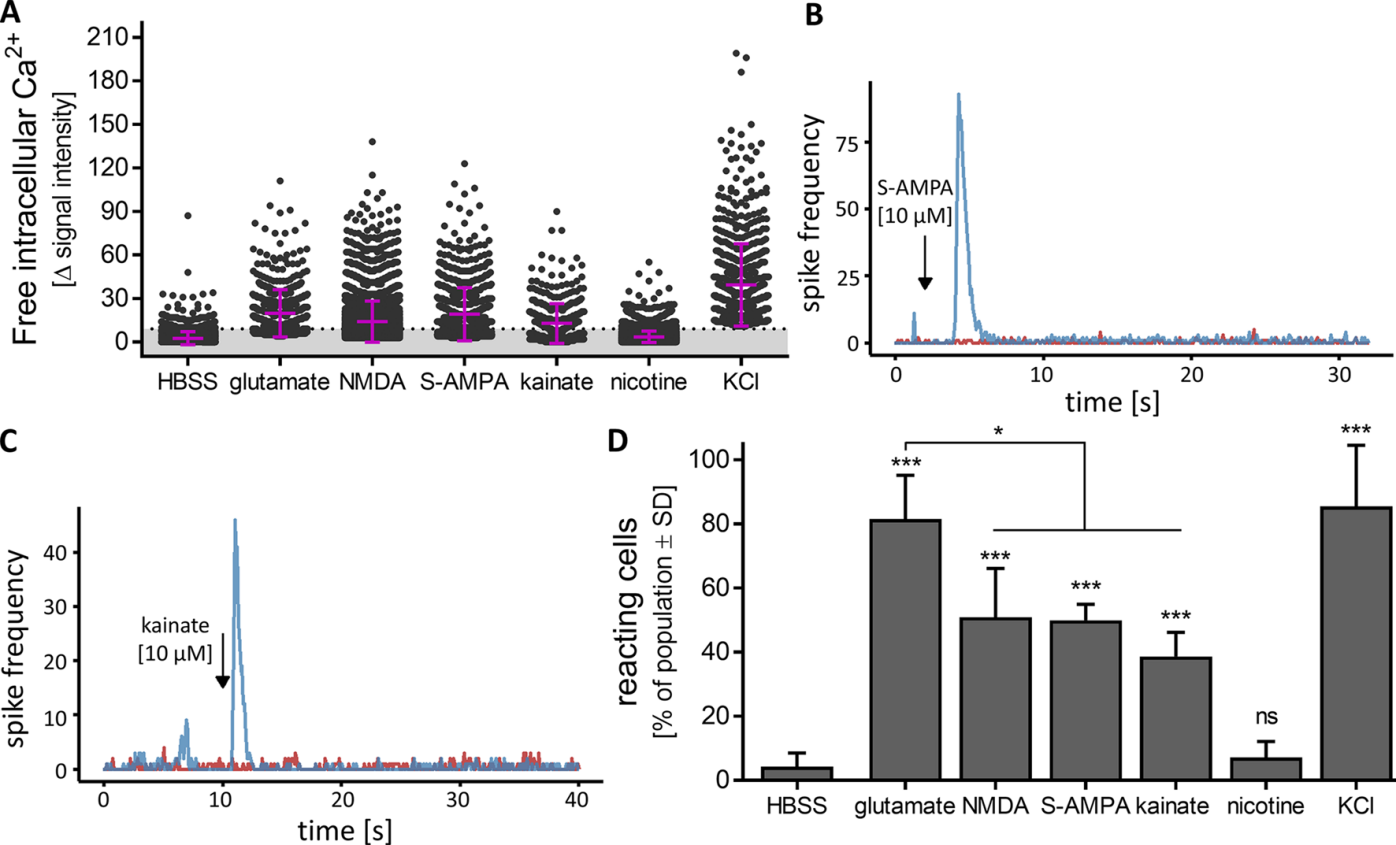
Characterization of different ionotropic glutamate receptors in MCCs. MCCs were differentiated for 24 days and subsequently used for Ca^2+^ signalling or MEA analysis. Agonists of different ionotropic glutamate receptors were applied. **a** The changes of Ca^2+^ indicator fluorescence (= delta signal intensity) of each individual cell treated with the negative control HBSS, nicotine [50 µM], the glutamate receptor agonists glutamate [10 µM], NMDA [50 µM], S-AMPA [10 µM], kainate [10 µM] or KCl [30 mM] is shown as a dot. The mean and SD is shown in purple. The threshold of Δ signal intensity ≥ 9 (= defined as reactive cell) is shown as dotted line. On DoD24 MCCs on MEAs were treated with **b** S-AMPA [10 µM], **c** kainate [10 µM] (blue, addition is indicated by the black arrow; baseline in red). The generation of spikes was recorded directly before and after administration and the number of spikes was binned (bin size 0.1 s). A respective example of the acute response is shown. **d** The data from (**a**) are shown as the percentage of reactive cells from the whole population. ***p value < 0.001, *ns*  not significant (color figure online)

To further characterize ionotropic glutamate receptors, using this optimized quantification protocol, cells were treated with glutamate [10 µM], KCl [30 mM], and NMDA [50 µM]. The positive responses of the positive control were confirmed. The negative control nicotine showed the expected (non)-response (Fig. 6a). In this broader test panel, we also included AMPA [10 µM], a stimulus of a subgroup of non-NMDA glutamate receptors (Hollmann and Heinemann 1994). The Δ signal intensity values indicated a clear response of MCC (Fig. 6a). This was confirmed using MEA technology where the addition of AMPA led to an immediate, transient increase in spike frequency (Fig. 6b). We also used kainate [10 µM], a seizurogenic compound triggering another subgroup of glutamate receptors (Hollmann and Heinemann 1994). The reaction to this agonist was slightly less intense but still, MCCs showed Δ signal intensity values significantly above the threshold (Fig. 6a), and MEA recordings confirmed the reactivity of the MCC network to kainate (Fig. 6c). A quantitative comparison of the percentage of reacting cells showed that glutamate was the most effective agonist, but still about half of MCCs reacted to each of the receptor subfamily agonists NMDA, AMPA and kainate (Fig. 6d). The heterogeneity of MCCs reflects the multiple cell types with their individual receptor patterns, as it is also common in human brains. The broad spectrum of glutamate responses allows for studies of activated or disturbed neuronal signalling not possible in frequently used human cell-based test systems, such as LUHMES cells (Gutbier et al. 2018; Lotharius et al. 2005; Scholz et al. 2011) or SH-SY5Y cells (Krebs et al. 2020).

### Toxicants acting on the glutamate signalling in MCC

**Fig. 7:**
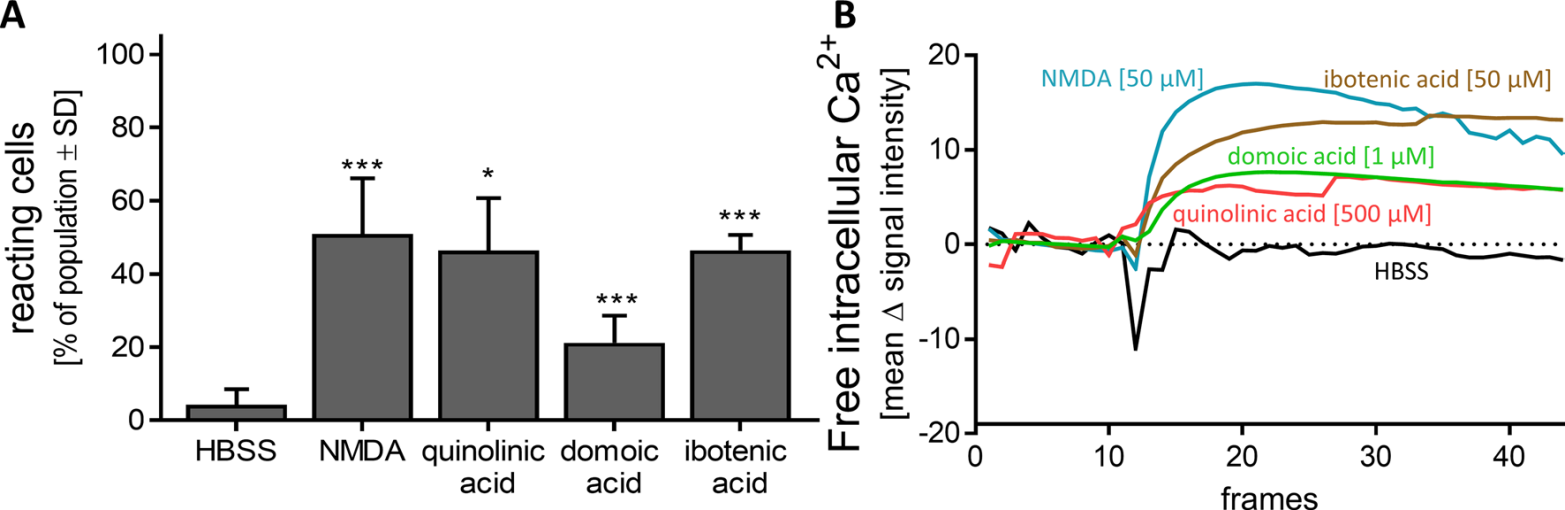
Characterization of NMDA receptor with agonists. DoD24 MCCs were used for Ca^2+^ imaging and were stimulated with different agonists of the NMDA receptor. **a** Percentage of cells reacting to HBSS, NMDA [50 µM], quinolinic acid [500 µM] (an endogenous NMDA-R agonist), domoic acid [1 µM] (a neurotoxin produced by algae, kainate- and AMPA-receptor agonist), or ibotenic acid [50 µM] (a fungal neurotoxin acting as NMDA-R agonist) compared to the whole cell population is shown. Data are means of three biological replicates ± SD. *p value < 0.05, ***p value < 0.001; **b** Averaged traces from Ca^2+^ signalling of **a** are shown. The full set of data with standard deviations is displayed in Fig. S6

The MCC system was established to measure glutamate responses in human neurons, and preferentially those mediated by the NMDA-R. Therefore, further agonists of the NMDA-R were investigated, and the percentage of reacting cells was determined: quinolinic acid is an endogenous NMDA-R agonist produced by microglia and macrophages in the kynurenine pathway of liver and brain. It is involved in many neurological diseases (Myint 2012). About 45% of MCCs reacted after quinolinic acid [500 µM] application (Fig. 7a). The same percentage of cells could be activated by the addition of ibotenic acid [50 µM] (Fig. 7a). This NMDA-R agonist, typically produced by, e.g. *Amanita muscaria* mushrooms, is often used for toxic lesioning models in rodents, and as a template to design new drugs (Krogsgaard-Larsen et al. 1996; Winn et al. 1984)*.* From this set of experiments, we conclude that neurotoxicants acting by NMDA-R agonism can be detected in MCCs.

As a further well-studied neurotoxicant, we chose domoic acid. This compound is produced by algae, and it accumulates in the marine food chain (Doucette and Tasker 2016). It is notorious for causing serious shellfish poisoning, and extensive pharmacological studies have identified it as a kainate receptor agonist (Larm et al. 1997). The percentage of reacting cells after administration was about 20% (Fig. 7a). It was striking, that the shape of Ca^2+^ imaging traces differed between the neurotoxins. The positive control NMDA [50 µM] showed a fast and steep increase in Δ signal intensity and a slow decrease after reaching the maximum. In contrast, the [Ca^2+^]_i_ response of ibotenic acid, domoic acid, and quinolinic acid increased slower but then remained at its highest level for at least 45 s. (Fig. 7b and Fig. S6A, B). This kinetic may be further explored in the future. While some of the observed differences may be related to receptor binding (off-rates) properties of the toxicants, they may also be caused by differential signalling (involvement of different Ca^2+^ pools, different secondary regulations, different desensitization of involved receptors). The later phase of the recorded Ca^2+^ kinetics may also involve intercellular processes, such as the activation of neighbouring cells to release neurotransmitters, which affect [Ca^2+^]_i_.

### Toxicity by glutamate receptor antagonism

**Fig. 8:**
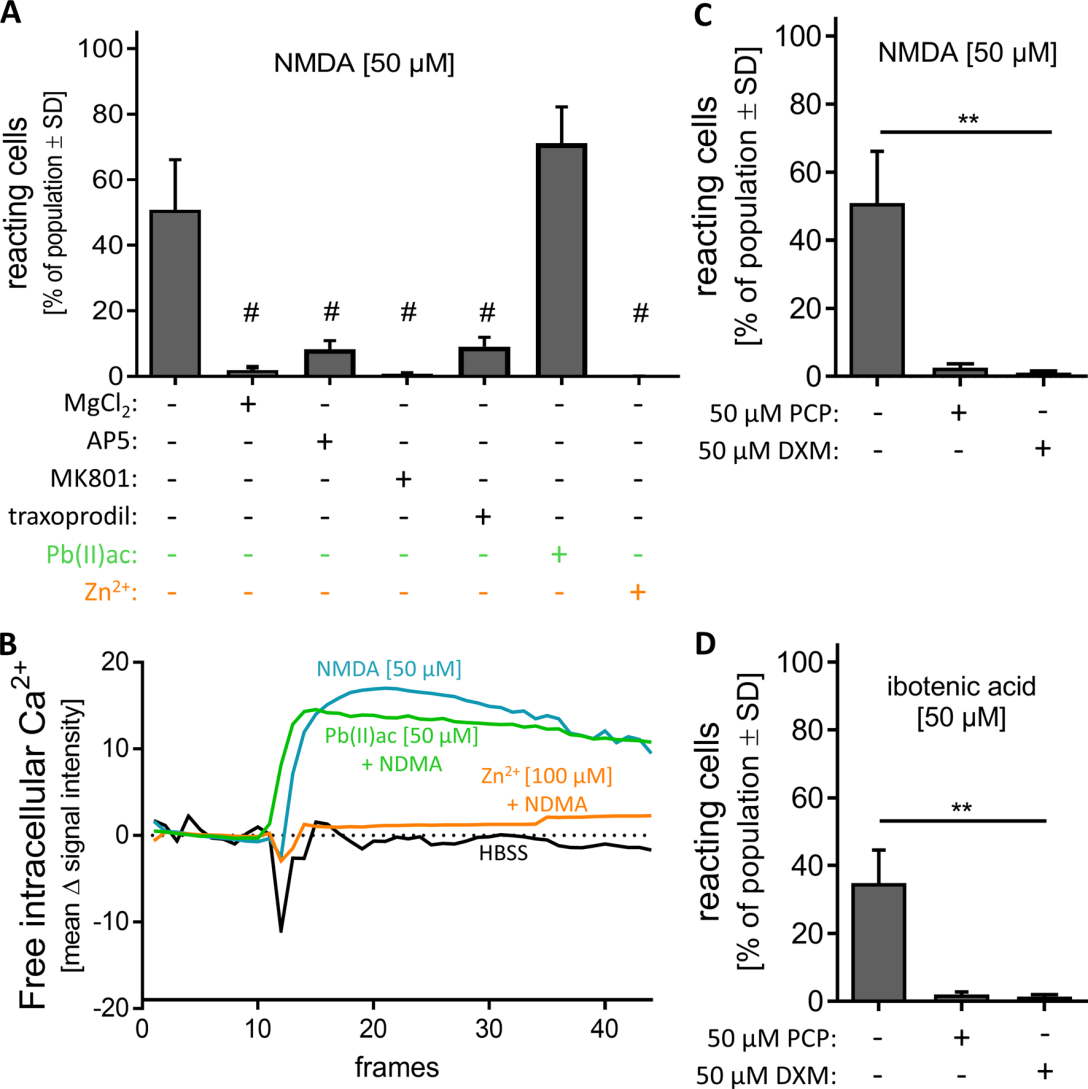
Characterization of NMDA receptor with antagonists. DoD24 MCCs were used for Ca^2+^ imaging and were preincubated with different antagonists of the NMDA receptor. **a** MCCs were pre-incubated for 30 min with different antagonists of the NMDA-R: MgCl_2_ [5 mM], AP5 [50 µM], MK801 [6 µM], traxoprodil [5 µM], Pb(II)ac [50 µM], or Zn^2+^ [10 µM] followed by addition of 50 µM NMDA. The percentage of reacting cells from the whole cell population is shown. Data are means of three biological replicates ± SD, #*p* value < 0.05 (reaction vs NMDA alone). **b** Averaged traces from Ca^2+^ signalling of **a**. The full set of data with standard deviations is displayed in Fig. S6. **c** MCCs were pre-incubated for 30 min with phencyclidine [50 µM] (PCP) or dextromethorphan [50 µM] (DXM) followed by the addition of NMDA [50 µM]. The percentage of reacting cells compared to the whole cell population is shown. Data are means of three biological replicates ± SD. **p value < 0.01. **d** MCCs were pre-incubated for 30 min with phencyclidine [50 µM] (PCP) or dextromethorphan [50 µM] (DXM) followed by the addition of ibotenic acid [50 µM]. The percentage of reacting cells compared to the whole cell population is shown. Data are means of three biological replicates ± SD; **p value < 0.01

We explored a panel of antagonists of the NDMA-R. First, we used apparently simple Mg^2+^ ions. As they block the NMDA-R pore channel, they can act as potent and efficient antagonists in cell culture experiments (Volbracht et al. 2006). When MCCs were pre-incubated with MgCl_2_ [5 mM] the response to NMDA [50 µM] was completely blocked (Fig. 8a). The antagonist MK801 (= dizocilpine) also acts on the channel pore and thereby blocks the NMDA-R (Scatton 1993). Indeed, no stimulation with NMDA [50 µM] was possible after pre-incubation with MK801 [6 µM] (Fig. 8a). When we used a classical competitive antagonist of the glutamate binding site, e.g. AP5, the NMDA response was attenuated by about 80% (Fig. 8a). As a further antagonist, we used traxoprodil [5 µM]. This compound specifically inhibits the NR2B subtype of NMDA-Rs. Like AP5 it reduced the NMDA response by about 80% (Fig. 8a). Traxoprodil was developed as neuroprotective agent to prevent brain damage after stroke (Wang and Shuaib 2005). Recently it has been considered as potential antidepressant (Preskorn et al. 2008).

After showing that well-established NMDA-R antagonists may be detected by our system, we investigated whether lead ions showed any effect. This is interesting as lead is a well-known developmental neurotoxicant, which changes synaptogenesis and NMDA-R behaviour (Toscano and Guilarte 2005). Older reports have suggested lead to be an antagonist or a negative allosteric modulator of the NMDA-R (Guilarte et al. 1995; Schulte et al. 1995). MCCs were pre-incubated with Pb(II)acetate [50 µM] prior to NMDA [50 µM] administration. The percentage of reacting cells was not significantly changed in the presence of lead (Fig. 8a). As positive control, cells were pre-incubated with Zn^2+^ [10 µM], a well-established negative regulator of the NMDA-R (Jalali-Yazdi et al. 2018). Indeed, we found that the NMDA-R could not be stimulated by NMDA anymore (Fig. 8a, b and Fig. S6D) when Zn^2+^ was present. The different effects of Pb^2+^ vs Zn^2+^ were fully confirmed when the entire traces generated by Ca^2+^ imaging were considered (Fig. 8b and Fig. S6C). These effects are in line with more recent literature on lead: it has become clear that the neurodevelopmental toxicity of lead is due to this metal ion interfering with synaptogenesis. Direct inhibition of the NMDA-R could not be confirmed. It is now rather assumed that Pb^2+^ affects synaptic signalling indirectly by attenuating BDNF transcription (Sachana et al. 2018). When lead affects BDNF in the developing CNS, synapses cannot form properly. Therefore, the vulnerability to lead exposure is higher in earlier developmental stages (Guilarte and Miceli 1992). Chronic Pb^2+^ exposure during development results in decreased levels of NR2A (Zhang et al. 2002). Although lead is a well-known DNT compound with severe effects on the nervous system, these are not acute effects and need time to establish. Here, the lead exposure was only 30 min and we only investigated direct effects on Ca^2+^ signalling. Thus, the absence of an inhibitory effect is fully consistent with the available literature.

The last group of compounds we explored was drugs, which may be used recreationally (illicitly) because of their psychedelic effects. We chose phencyclidine (PCP) and dextromethorphan (DXM) to investigate their effect on the NMDA-R in MCCs. Both showed a strong antagonistic effect, and they fully abolished the Ca^2+^ response after stimulation with either NMDA [50 µM] (Fig. 8c) or ibotenic acid [50 µM] (Fig. 8d). The cough suppressant DXM binds like ketamine and PCP to the dizocilpine site of the NMDA-R. All three substances are abused as illicit drugs inducing psychoses (Jodo 2013; Martinak et al. 2017; Powers et al. 2015) and symptoms of schizophrenia (Frohlich and Van Horn 2014; Jodo 2013). These symptoms are associated with the NMDA-R antagonistic effects of the compounds. Our MCC-based test system may be used to identify such compounds from various sources (drugs, environmental toxicants, and natural occurring neurotoxins).

## Conclusions and outlook

In this study, we presented a test system comprised of excitatory and inhibitory neurons as well as glial cells. We showed that [Ca^2+^]_i_ imaging can be used as a quantitative readout despite the heterogeneity of cell types present in MCC. The functional glutamate receptors were further characterized as belonging to the subclasses of NMDA-R, AMPA receptors, and kainate receptors. We focused on NMDA-R responses by applying various agonists and antagonists.

As NMDA-R have such a particularly high physiological, pathological and pharmacological importance, several other differentiation protocols for neurons containing functional NMDA-R have been developed in the past (Heikkilä et al. 2009; Meijer et al. 2019; Nehme et al. 2018; Pruunsild et al. 2017; Yamazaki et al. 2016). The objective of these studies was to examine the role of NMDA-R for biological functions (gene expression, synaptic transmission and plasticity, modulation by astrocytes). They did not focus on toxicological testing and usually provided little information on endpoint quantification. To our knowledge, neither a response profiling by agonists and antagonists, nor a clear description of test system responses on the population level is available elsewhere.

Most cell systems developed for neurotoxicity testing have until recently been based on rodent primary cultures (Alépée et al. 2014; Hogberg et al. 2011; Hondebrink et al. 2016; Kosnik et al. 2020; Kreir et al. 2018; Strickland et al. 2018; Suñol et al. 2008; Zurich et al. 2013; Zwartsen et al. 2018). The human iPSCs based systems that have undergone first evaluations rely on commercially available cells with costs that prevent their routine use in academic laboratories. Moreover, the first data from such systems did not provide strong evidence for a robust NMDA-R response. However, compounds acting on other glutamate receptors like domoic acid, have shown clear responses (Nimtz et al. 2020; Tukker et al. 2020). As MCCs can be generated relatively fast, and Ca^2+^ imaging is performed in 96-well format, our system allows a sufficient throughput for small screens and relatively extensive follow-up characterizations.

During system evaluation, we also considered another important pathological process related to NMDA-R: excitotoxicity (Choi et al. 1989; Leist et al. 1997a, 1998; Olney and de Gubareff 1978). However, we did not observe cell death responses after exposure to glutamate or NMDA for up to 24 h. However, this may be related to the medium composition or other culture factors that are modifiable, and the issue deserves further investigation in the future.

One of the most comprehensive approaches to identify potential functional neurotoxicity is the use of neuronal networks on MEA (Kosnik et al. 2020; Strickland et al. 2018; Vassallo et al. 2017). The hitherto most robust MEA data have been generated with rat primary neurons. More recently, murine and human-stem cell-derived neurons have been demonstrated to be principally suitable for the analysis of network signalling on MEA (Pagan-Diaz et al. 2020; Tukker et al. 2020). However, the production of cultures at a reasonable price, time effort and robustness has proven challenging. Under such conditions, the presence of both, excitatory and inhibitory neurons is important to obtain stable networks (Tukker et al. 2018; Zou et al. 2020). We have shown for MCC that they may be used for such MEA analysis. They develop a functional neuronal network within 24 days. This is relatively fast compared to other differentiation protocols based on PSC (Cao et al. 2017; Klapper et al. 2019; Meijer et al. 2019; Nehme et al. 2018; Russo et al. 2018). Our initial MEA data (NMDA/bicuculline exposure) suggest that both excitatory and inhibitory neurons are present. Additionally, MCCs contain glial cells, which are essential for synaptogenesis and maturation of a functional neuronal network (Ishii et al. 2017; Klapper et al. 2019). As MEA analysis was not the focus of the current work, mostly qualitative and descriptive data have been presented, but the speed of differentiation and the strength of the responses observed seem promising for further adaptation of MCC to MEA.

We focused here on toxicological applications, as neurotoxicity is one of the major reasons for drug failure (Cook et al. 2014). However, MCC may be interesting also for general biomedical research. As many types of receptors are expressed, it may be possible to use the system to investigate multiple complex network responses, such as seizurogenic activity or circuitry disturbances related to schizophrenia.

## Supplementary Information

Below is the link to the electronic supplementary material.

Supplementary file1 (PDF 1823 KB)

Supplementary file2 (XLSX 377 KB)

## Abbreviations

AMPA: α-Amino-3-hydroxy-5-methyl-4-isoxazolepropionic acid
AOP: Adverse outcome pathway
BDNF: Brain-derived neurotrophic factor
BIC: Bicuculline
[Ca^2^^+^]_i_: Intracellular free concentration of Ca^2^^+^ ions
cAMP: Cyclic adenosine monophosphate
CNS: Central nervous system
DEG: Differentially expressed gene
DMSO: Dimethyl sulfoxide
DoD: Day of differentiation
DXM: Dextromethorphan
EB: Embryoid body
FC: Fold change
GDNF: Glial cell-derived neurotrophic factor
Glu: Glutamate
GO: Gene ontology
hESC: Human embryonic stem cell
hiPSC: Human induced pluripotent stem cell
ISI: Inter spike interval
KBP: Key biological process
MCC: Mixed cortical culture
MEA: Microelectrode array
MK801: Dizocilpine
NESC: Neuroepithelial stem cells
NMDA: *N*-Methyl-d-aspartate
NMDA-R: *N*-Methyl-d-aspartate receptor
oGO: Overrepresented gene ontologies
PC: Principal component
PCA: Principal component analysis
PCP: Phencyclidine
PMA: Purmorphamine
RT-qPCR: Real-time quantitative polymerase chain reaction
SD: Standard deviation
TPM: Transcripts per kilobase million
