# Supplementary material for: A human stem cell-derived test system for agents modifying neuronal N-methyl-d-aspartate-type glutamate receptor Ca2+-signalling
Source: Arch Toxicol. 2021 Mar 13;95(5):1703–22. doi: 10.1007/s00204-021-03024-0 (PMC8113295; doi:10.1007/s00204-021-03024-0)
Supplement: Supplementary file 1 — Supplementary file1 (PDF 1823 KB) [file 204_2021_3024_MOESM1_ESM.pdf]

Supplementary information for

A human stem cell-derived test system for agents modifying neuronal N-methyl-D-aspartate-type glutamate receptor Ca^{2+}-signalling

Stefanie Klima[1,2], Markus Brüll[1], Anna-Sophie Spreng[1,3], Ilinca Suciu[1,3], Tjalda Falt[1], Jens C Schwamborn[4], Tanja Waldmann[1], Christiaan Karreman[1], Marcel Leist[1,5]

Table of Contents

Tab. S1	P2	Overview of compounds used in this study
Tab. S2	P2	Primary antibodies used in this study
Tab. S3	P3	Primers used in this study
Tab. S4	P3	Numeric values of log_2 fold change transcriptome data
Fig. S1	P6	Characterization of NESC
Fig. S2	P8	Characterization of MCCs at different developmental stages
Fig. S3	P10	Comparison between transcriptome analysis of ESC-derived and iPSC-derived MCCs
Fig. S4	P12	Determination of reactivity threshold
Fig. S5	P13	Specificity controls of Ca^{2+} signaling in MCCs and peripheral neurons
Fig. S6	P14	Ca^{2+} traces of MCCs to characterize their NMDA receptor

Supplementary table 1: Overview of compounds used in this study

Compound	Solvent	Concentration [µM]	Supplier	Catalogue number
AP5	water	50	Sigma	A8054
bicuculline	DMSO	1	Sigma	B-9130
dextromethorphan (DXM)	water	50	Sigma	D2531
domoic acid	water	1	Sigma	D6152
glutamate	water	0.5-20	Sigma	G1626
ibotenic acid	water	50	Tocris	0285
kainate	water	10	Tocris	0222
KCl	water	10000, 30000	Sigma	P9541
ketamine	water	50	Sigma	K2753
$MgCl_2$	water	5000	Merck	105833
MK801	DMSO	6	Sigma	M107
nicotine	DMSO	50	Sigma	36733
NMDA	water	0.5-50	Tocris	0114
Pb(II)ac	water	50	Sigma	316512
phencyclidine (PCP)	water	50	Tocris	2557
quinolinic acid	DMSO	500	Sigma	P63204
S-AMPA	water	10	Tocris	0254
traxoprodil	DMSO	5	Sigma	SML0053
veratridine	DMSO	100	alomone labs	V-110
Zn^{2+}	water	100	Sigma	Z0152

Supplementary table 2: Primary antibodies used in this study

Target	Isotype	Dilution	Supplier	Catalogue number	Lot number
TUBB3	mouse IgG2a	1:1000	Covance	MMS-435P	D13AF00117
Nestin	mouse IgG1	1:100	R&D	MAB1259	HSG0213091
Ki67 (PE)	mouse IgG1	1:600	BD Pharmingen	556027	03428
DCX	goat	1:100	Santa Cruz	sc-8067	L1008
PAX6	rabbit	1:200	Covance	PRB-278-P	D14BF00330
PAX3	mouse IgG2a	1:200	Ch. O. Ordahl, San Francisco		
SOX1	goat	1:200	R&D Systems	AF3369	XUV0213111
MAP2	mouse IgG1	1:1000	Sigma	M4403	063M4802
NeuN	mouse IgG1	1:200	Merck Millipore	MAB377	2159655
Gad65/67	rabbit	1:200	Bioworld	BS1400	360803
Synaptophysin	mouse IgG1	1:200	Synaptic Systems	101011	1-51
S100ß	mouse IgG1	1:500	Sigma	S2532	023M4828
vGlut1	rabbit	1:500	Synaptic-Systems	AB5905	LV1439669

Supplementary table 3: Primers antibodies used in this study

Target	Sequence (For)	Sequence (Rev)
CDK1	AGAGCTTTGGGCACTCCCAA	TGCCAGAAATTCGTTTGGCTG
CDK2	GAATCTCCAGGGAATAGGCC	TACACCCATGAGGTGGTGAC
CYCLIN D1	CTCACACGCTTCCTCTCCA	CACCTCCTCCTCCTCCTCTT
CYCLIN D2	ATGTTCCTGGCCTCCAAAC	GCCAGGTTCCACTTCAACTT
DCX	GCGAAATTTTTCAGGACCAC	CACAGAAGCCATCAAACTGG
GAPDH	ATGGAGAAGGCTGGGGCTCA	AGTGATGGCATGGACTGTGGTCAT
GFAP	CGAGATCGCCACCTACAGGAAGC	CCTTAATGACCTCTCCATCCCGC
HES5	TTGGAGTTGGGCTGGTG	CCCAAAGAGAAAAACCGA
MAP2	CGGATGACCTAAAGCGATTC	TCTGGAGCATTGTCATTTGC
NANOG	GGTGAAGACCTGGTTCCAGAAC	CATCCCTGGTGGTAGGAAGAGTAAAG
NESTIN	GCACCTCAAGATGTCCCTCAGC	GGGAAGTTGGGCTCAGGACTG
NEUN	AGCCCGGGAGAAGCTGAATG	GTGCCGGTGGTGGGGTAGGG
NR1	GTCCACCAGACTGAAGATTGTGAC	CTCCTCCTTGCATGTCCCA
NR2A	GCTCTTCTCCATCAGCAGGG	GGATCCCGTCAGATTGAAGTCT
NR2B	GGTCTTCTCCATCAGCAGAGG	TGTTGTTCATGGTTGCGGT
OCT4	GCAAAGCAGAAACCCTCGTGC	ACACTCGGACCACATCCTTCTCG
PAX3	GACTGGCTCCATACGTCCTGGTGC	CGGCTGATGGAACTCACTGACGG
PAX6	CCGCCTATGCCCAGCTTCAC	AAGTGGTGCCCGAGGTGCCC
PSD95	CACTCCTCACAGTGCTGCAT	TGTCTTCATCTTGGTAGCGG
RPL13A	GGTATGCTGCCCCACAAAACC	CTGTCACTGCCTGGTACTTCCA
S100ß	CTTAGAGGAAATCAAAGAGCAGGAGGT	CATGTTCAAAGAACTCGTGGCAGG
SLC17A6	GCGGCCTGGGCTTCTGCATC	AGCCGAGACGCGATGTAGCC
SOX1	CCGGGATAAGGGCCTCCCCA	ACACAGGGGAGCACAGGGGC
SYNAPSIN	TCAGACCTTCTACCCCAATCA	GTCCTGGAAGTCATGCTGGT
TBP	GGGCACCACTCCACTGTATC	GCAGCAAACCGCTTGGGATTATATTCG
TUBB3	AACTACGTGGGCGACTCGGA	GTTGTTGCCGGCCCCACTCT

Supplementary table 4: Numeric values of log_2 fold change transcriptome data

Neuroepithelial stem cells (NESCs) from two pluripotent stem cell lines (H9 and Sigma iPSC) were differentiated into mixed cortical cultures (MCCs), and mRNA was prepared for transcriptome analysis. From a set of about 3500 genes the expression of selected neuronal, non-neuronal and stem cell markers for two cell lines is shown in a heatmap in Fig 2B. The data are given on a log_2 fold change scale with MCC relative to NESC. Here the log_2 fold changes (log2FC), adjusted p-values (padj) and the standard errors of log2FC (lfcSE) are given for all genes included in the heatmap.

Gene name	logFC		padj		lfcSE	
	Sigma	H9	Sigma	H9	Sigma	H9
DCX	3.83	3.34	2.17E-78	4.74E-19	0.20	0.36
TUBB3	0.40	0.33	1.20E-01	5.74E-01	0.23	0.45
MAP2	2.63	2.53	7.49E-59	1.00E-36	0.16	0.19
NEFL	0.97	3.12	7.46E-05	1.21E-15	0.23	0.37
NEFH	-2.10	0.31	3.44E-16	7.40E-01	0.25	0.70
APP	2.62	2.17	2.77E-21	1.13E-06	0.27	0.42
MAPT	6.01	5.85	3.62E-196	1.40E-45	0.20	0.40
RBFOX3	2.58	5.56	1.86E-06	2.05E-11	0.52	0.79
SNCA	1.05	2.92	1.45E-04	3.86E-20	0.26	0.31
ENO2	3.68	3.15	2.88E-96	3.36E-27	0.17	0.28
PRNP	0.77	1.65	1.20E-04	2.19E-04	0.19	0.41
DLG4	2.58	2.44	5.66E-85	3.76E-30	0.13	0.21
HOMER1	0.80	0.68	1.13E-03	9.99E-02	0.23	0.35
HOMER2	-0.92	0.78	6.14E-03	4.64E-02	0.32	0.34
SNAP25	2.98	3.31	1.46E-29	9.25E-16	0.26	0.39
SYN1	3.24	4.81	3.58E-34	7.37E-34	0.26	0.39
SYP	0.50	4.49	1.98E-01	1.62E-12	0.34	0.61
GRIA2	7.88	6.05	6.33E-103	8.94E-46	0.36	0.41
GRIA3	3.89	5.17	2.49E-28	3.56E-16	0.35	0.61
GRIK4	4.37	4.60	3.07E-35	1.79E-13	0.35	0.60
GRIK5	5.57	4.94	5.11E-23	3.81E-18	0.55	0.55
GRIN2A	0.90	4.82	2.31E-01	1.85E-04	0.66	1.18
GRIN2C	-1.07	-1.40	4.81E-02	7.21E-02	0.50	0.66
GRIN2D	4.28	6.15	3.21E-15	4.75E-09	0.53	0.99
GRM4	4.97	6.09	9.56E-10	2.04E-08	0.79	1.02
GRIN1	6.60	7.92	1.65E-20	7.32E-14	0.70	1.01
GRIN2B	3.30	9.68	2.51E-24	2.56E-23	0.32	0.94
GLUL	-2.14	-0.43	3.72E-19	2.19E-01	0.23	0.29
SLC17A6	7.67	8.05	2.32E-126	1.57E-58	0.32	0.49
SLC17A7	0.26	1.98	NA	NA	3.06	1.96
GABRA2	7.88	7.63	5.16E-22	1.53E-11	0.80	1.08
GABRB3	3.38	2.92	2.22E-21	3.23E-26	0.35	0.27
GABBR1	1.45	1.78	1.88E-09	8.96E-07	0.23	0.34
GAD1	0.64	4.88	4.14E-02	2.44E-13	0.29	0.64
GAD2	4.63	7.22	6.11E-24	1.29E-10	0.45	1.07
NES	-3.89	-1.85	5.20E-29	4.04E-06	0.34	0.37
VIM	-0.14	-0.50	4.34E-01	NA	0.15	0.43
AQP4	2.94	2.33	8.02E-08	2.52E-01	0.53	1.65
GFAP	10.53	-0.41	4.72E-39	NA	0.79	2.02
SLC1A2	0.84	1.76	2.51E-02	3.09E-07	0.35	0.32
SLC1A3	2.70	2.31	2.74E-19	2.55E-05	0.29	0.51
S100B	1.72	6.30	7.04E-07	3.84E-24	0.34	0.60
MAOB	3.23	3.20	4.57E-22	3.76E-04	0.33	0.82
MBP	-1.18	0.50	6.90E-01	NA	2.37	3.17
OLIG2	-4.56	3.09	2.33E-21	6.73E-02	0.47	1.45

CYP3A4	-1.93	-0.82	5.69E-01	NA	2.79	3.19
CYP1A2	-1.18	-0.24	NA	NA	3.06	3.19
GSTA1	-1.43	-1.90	1.44E-01	2.94E-01	0.88	1.47
GSTA2	-1.54	-1.14	6.54E-01	NA	2.77	2.31
CYP2E1	3.08	2.10	2.61E-08	8.54E-04	0.54	0.57
CYP2C8	1.88	1.20	9.89E-02	4.19E-01	1.03	1.17
SULT2A1	NA	-0.24	NA	NA	NA	3.19
SLCO1B1	-0.46	-1.18	NA	NA	3.06	3.19
SOX17	-0.82	0.48	7.89E-01	NA	2.48	3.18
FOXD3	-0.49	1.97	2.09E-01	NA	0.35	1.94
MSX1	-1.28	-1.86	4.82E-05	9.24E-03	0.30	0.63
NGFR	-0.01	0.92	9.65E-01	9.08E-02	0.22	0.46
PAX3	-2.25	-2.63	9.18E-15	1.47E-06	0.28	0.51
ROBO1	3.35	2.07	3.63E-30	5.22E-08	0.29	0.36
SOX10	-6.92	-0.65	2.54E-59	7.91E-01	0.42	1.81
SOX9	2.35	2.60	4.40E-13	5.48E-12	0.32	0.36
TWIST1	1.22	-0.46	5.90E-03	NA	0.42	2.75
SNAI2	3.05	2.22	4.02E-12	2.28E-03	0.43	0.66
HTR1B	0.63	2.16	8.39E-01	2.36E-01	2.48	1.49
HTR6	0.58	0.12	7.85E-01	NA	1.73	3.19
HTR5A	1.96	3.84	4.85E-02	1.01E-02	0.91	1.32
ADRB2	-0.25	-0.65	8.49E-01	5.41E-01	1.05	0.82
DRD2	-0.31	3.03	2.61E-01	5.13E-11	0.24	0.44
KCNJ6	1.26	-0.41	6.69E-02	5.60E-01	0.63	0.54
NR4A2	5.31	3.39	2.59E-11	1.21E-06	0.77	0.65
SLC6A3	-0.48	-1.26	8.01E-01	5.15E-01	1.54	1.50
SLC18A2	1.48	1.47	3.64E-03	6.55E-02	0.48	0.68
TH	4.61	2.12	7.89E-14	1.87E-02	0.60	0.79
COMT	-1.51	0.47	7.91E-04	7.64E-01	0.43	1.16
CHRNA2	1.26	1.21	4.92E-01	6.67E-01	1.53	2.12
CHRNA4	2.00	2.51	2.71E-14	1.11E-05	0.26	0.53
CHRNB2	1.30	2.59	2.78E-06	6.71E-08	0.27	0.45
ACHE	4.54	6.92	1.30E-65	6.24E-45	0.26	0.48
CHAT	0.29	3.60	9.11E-01	2.81E-02	2.02	1.43
P2RY13	-1.54	NA	NA	NA	3.06	NA
P2RY14	NA	0.40	NA	NA	NA	2.64
P2RX3	3.19	1.90	1.33E-26	3.92E-07	0.29	0.35
CDH1	1.99	0.77	5.95E-02	7.32E-01	0.97	1.67
FUT4	-0.05	0.24	9.62E-01	8.41E-01	0.77	0.89
NANOG	-0.12	1.19	8.54E-01	2.74E-01	0.53	0.88
POU5F1	-2.54	0.12	8.23E-24	NA	0.25	3.19
SOX2	-0.81	-0.86	1.32E-05	1.67E-02	0.18	0.32

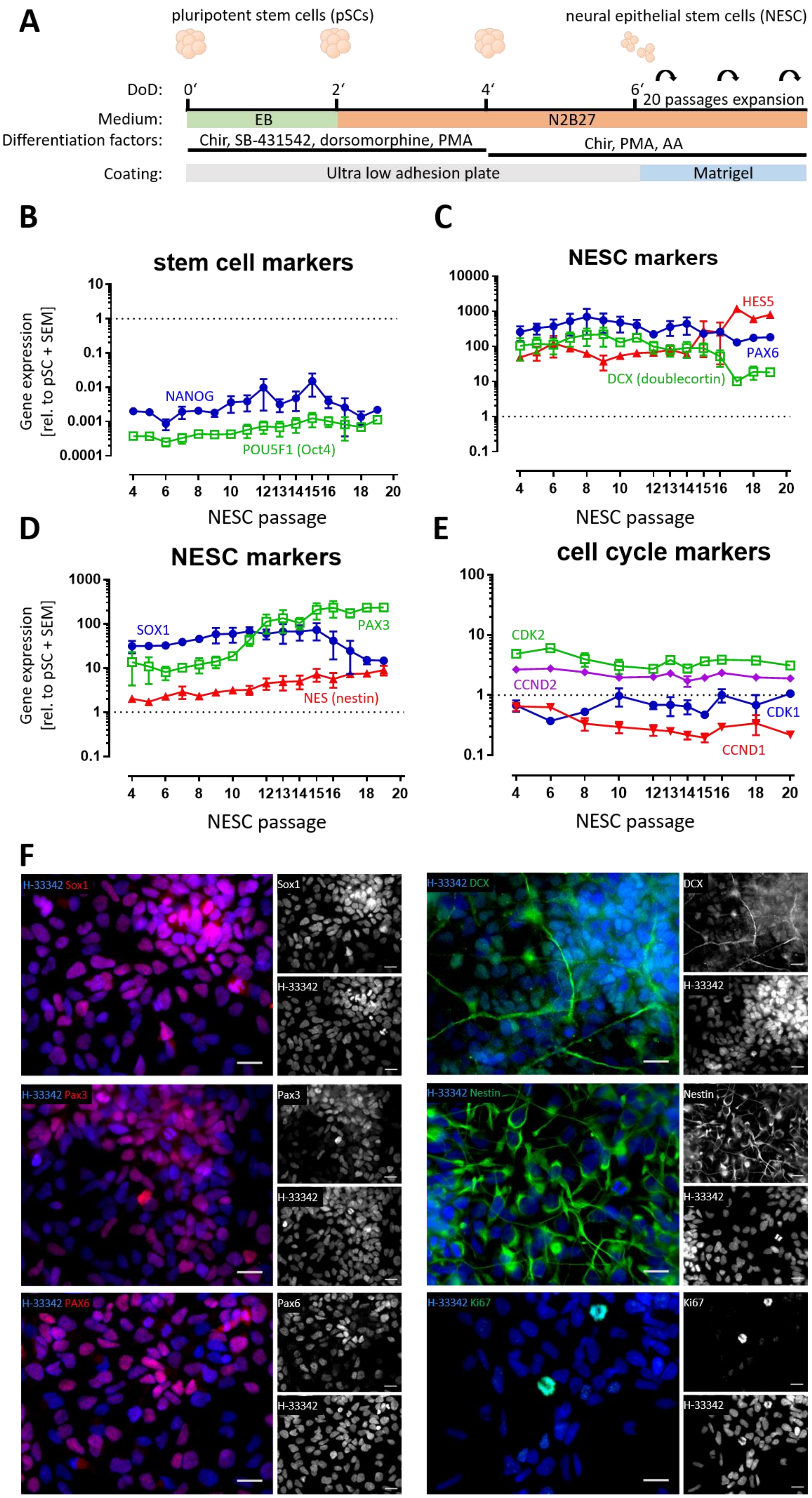

A
pluripotent stem cells (pSCs)
neural epithelial stem cells (NESC)
DoD:
0‘
2‘
4‘
6‘
20 passages expansion
Medium:
EB
N2B27
Differentiation factors:
Chir, SB-431542, dorsomorphine, PMA
Chir, PMA, AA
Coating:
Ultra low adhesion plate
Matrigel
B
stem cell markers
Gene expression [rel. to pSC + SEM]
NANOG
POU5F1 (Oct4)
NESC passage
C
NESC markers
HES5
PAX6
DCX (doublecortin)
NESC passage
D
NESC markers
Gene expression [rel. to pSC + SEM]
SOX1
PAX3
NES (nestin)
NESC passage
E
cell cycle markers
CDK2
CCND2
CDK1
CCND1
NESC passage
F
H-33342 Sox1
Sox1
H-33342
H-33342 DCX
DCX
H-33342
H-33342 Pax3
Pax3
H-33342
H-33342 Nestin
Nestin
H-33342
H-33342 PAX6
Pax6
H-33342
H-33342 Ki67
Ki67
H-33342

Supplementary figure 1: Characterisation of NESC

Neuroepithelial stem cells (NESC) were generated from pluripotent stem cells (pSCs) according to Reinhardt *et al.* (Reinhardt et al. 2013) by differentiating for 6 days in 3D spheres, followed by an expansion and maintenance phase of up to 20 passages. (A) Schematic display of NESC differentiation and expansion (Chir – Chir99021, PMA – purmorphamine, AA – ascorbic acid). (B) Gene expression profiles of the stem cell markers NANOG, POU5F1. Gene expression was quantified by real-time PCR. Data are shown relative to pSCs. (C and D) Gene expression of NESC markers PAX6, DCX, HES5, SOX1, PAX3, NES. Gene expression of the cell cycle markers cyclin dependent kinase CDK1, CDK2, CCND1, and CCND2. All data are means ± SEM of 2-3 biological replicates and cover NESC passage 4 until NESC passage 20. (F) Immunofluorescence images of NESC at passage 13 using antibodies against the neural progenitor markers Sox1, Pax3, Pax6, doublecortin (DCX), nestin, and the proliferation marker Ki67 (green). Nuclei were counterstained with Hoechst H-33342. Scale bars: 50 µm.

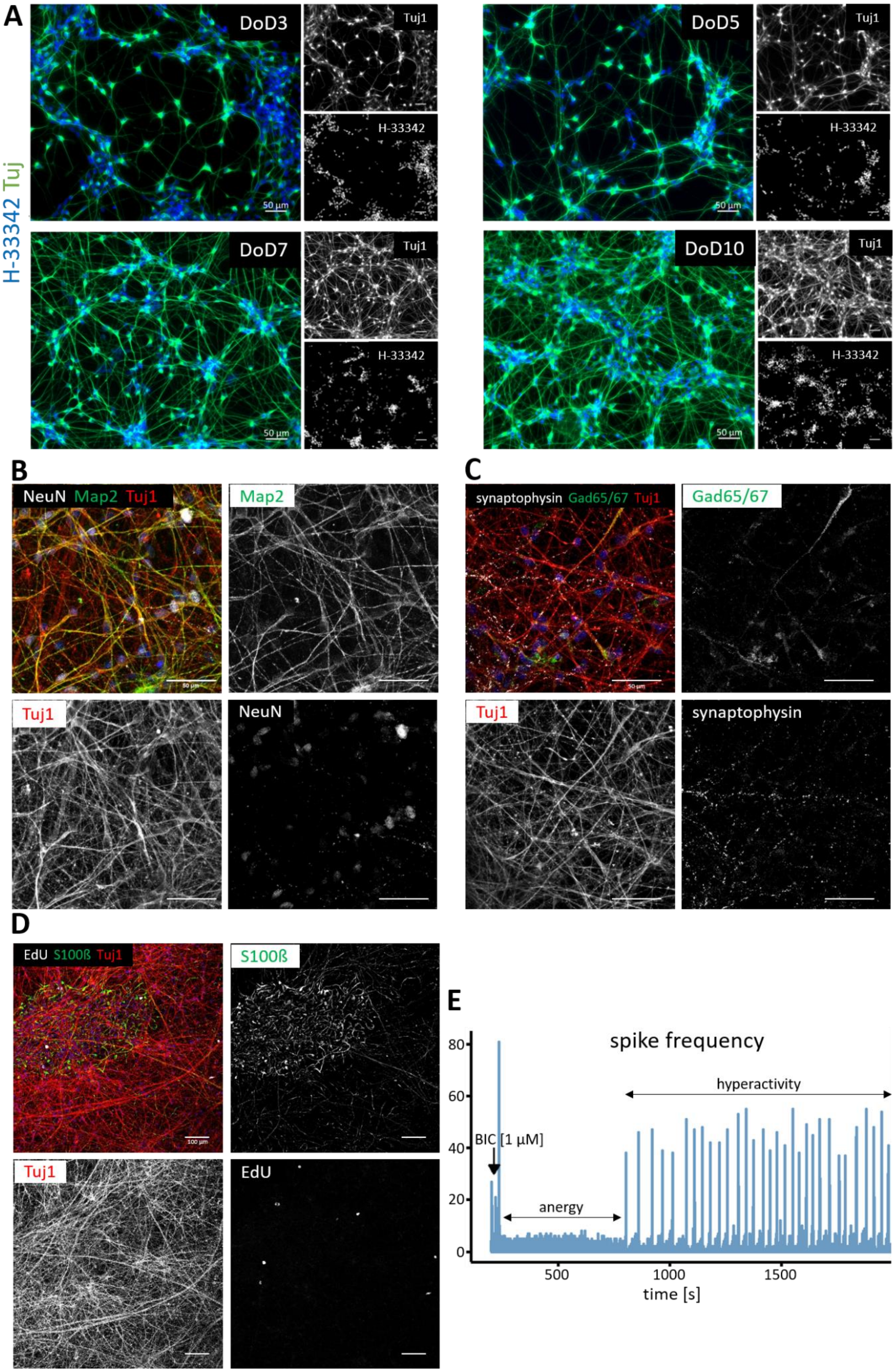
A
H-33342 Tuj
DoD3
Tuj1
H-33342
50 µm
DoD5
DoD7
DoD10
B
NeuN Map2 Tuj1
Map2
Tuj1
NeuN
C
synaptophysin Gad65/67 Tuj1
Gad65/67
Tuj1
synaptophysin
D
EdU S100ß Tuj1
S100ß
Tuj1
EdU
100 µm
E
spike frequency
hyperactivity
BIC [1 µM]
anergy
80
60
40
20
0
500
1000
1500
time [s]

Supplementary figure 2: Characterization of MCCs at different developmental stages

MCCs were differentiated on glass coverslips for 3, 5, 7, 10, or 24 days and fixed for immunostaining on the respective day. (A) Time course of neuronal network formation: cells stained on DoD3, DoD5, DoD7, and DoD10 with an antibody against the neuron specific cytoskeletal marker beta-III-tubulin (Tuj1). (B) Neurons were differentiated for 24 days and stained with markers of mature neurons like NeuN, Map2, and the neuronal marker Tuj1. (C) Immunostaining of the presynaptic marker synaptophysin, Gad65/67, the enzyme catalyzing the decarboxylation of glutamate to γ-aminobutyric acid (GABA), and the neuronal marker Tuj1 on DoD24. (D) Replicating cells on DoD24 were visualized by the nucleotide analog 5-ethynyl-2'-deoxyuridine (EdU), cells were counterstained against the glial cell marker S100ß, and the neuronal marker Tuj1. Scale bars: 50 µm. (E) MCCs were differentiated on MEA plates. Spontaneous spikes of electrical activity were recorded. On DoD24 MCCs on MEAs were treated with the $GABA_A$ receptor antagonist bicuculline [1 µM] (addition is indicated by a black arrow). The generation of spikes was recorded directly before and after administration, the number of spikes was binned (bin size 0.1 s) and a representative example of the response is shown.

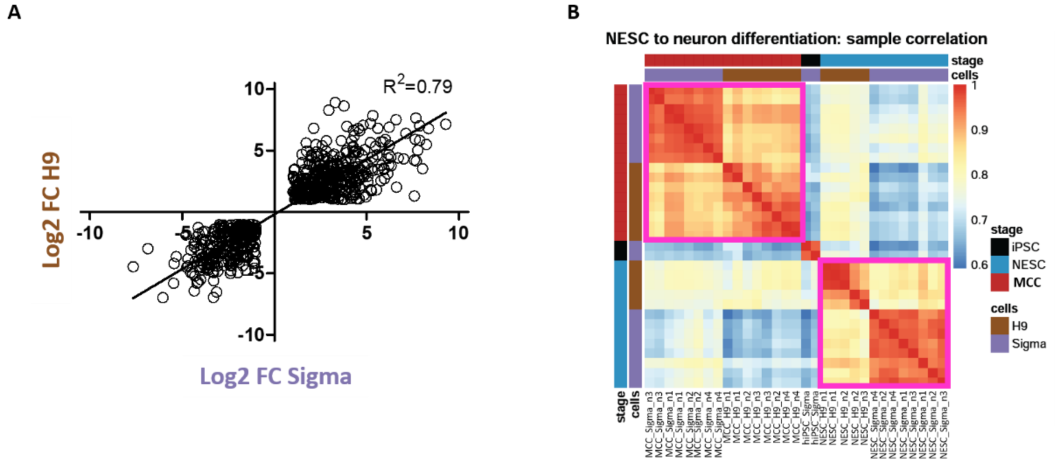

C

key biological process	term name	term id
neurodevelopment	regulation of nervous system development	GO:0051960
	cell morphogenesis involved in neuron differentiation	GO:0048667
	regulation of membrane potential	GO:0042391
	axon development	GO:0061564
	regulation of neurogenesis	GO:0050767
	axonogenesis	GO:0007409
	regulation of neuron differentiation	GO:0045664
	positive regulation of nervous system development	GO:0051962
	negative regulation of nervous system development	GO:0051961
	regulation of neuron projection development	GO:0010975
	negative regulation of neurogenesis	GO:0050768
	positive regulation of neurogenesis	GO:0050769
	positive regulation of neuron differentiation	GO:0045666
	regulation of axonogenesis	GO:0050770
neuronal subtype (glutamate)	regulation of NMDA receptor activity	GO:2000310
	glutamate receptor signaling pathway	GO:0007215
	regulation of glutamate receptor signaling pathway	GO:1900449
synapse	regulation of trans-synaptic signaling	GO:0099177
	synaptic signaling	GO:0099536
	trans-synaptic signaling	GO:0099537
	anterograde trans-synaptic signaling	GO:0098916
	neuron projection morphogenesis	GO:0048812
	regulation of postsynaptic membrane potential	GO:0060078
	positive regulation of synaptic transmission	GO:0050806
	regulation of synaptic plasticity	GO:0048167
	chemical synaptic transmission, postsynaptic	GO:0099565
neurotransmitter	modulation of chemical synaptic transmission	GO:0050804
	chemical synaptic transmission	GO:0007268
	neurotransmitter transport	GO:0006836
	regulation of neurotransmitter levels	GO:0001505
	regulation of neurotransmitter receptor activity	GO:0099601

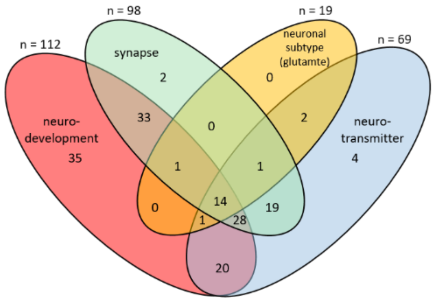

E

genes	fold change	comment
CCL2	43	not neurodevelopment; CCL2 inhibits glutamate-induced neuronal cell death
PPARGC1A	11	overlaps with neurotransmitter; interacts and regulated with CREB
KLHL24	9	overlaps with neurotransmitter; involved in regulation of kainate receptors
ACTN2	6	not in synapse; actinin alpha 2 belongs to the spectrin superfamily
GNAQ	2	not in neurotransmitter; G-protein subunit alpha Q

Supplementary figure 3: Comparison between transcriptome analysis of ESC-derived and iPSC-derived MCCs

Neuroepithelial stem cells (NESCs) from two pluripotent stem cell lines (H9 and Sigma iPSC) were differentiated into mixed cortical cultures (MCCs), and mRNA was prepared for transcriptome analysis. A set of about 3500 genes was used for targeted RNAseq analysis (TempOSeq method), and expression levels were obtained for four cell preparations (some with two samples per cell preparation). For both cell types differentially expressed genes (DEGs) between NESC and MCC were identified, for further analysis only DEGs present in both H9-derived and iPSC-derived cells were used. (A) Log2FC of DEGs from H9-derived and iPSC-derived cells were plotted against each other and the R^2 of the correlation was determined. (B) A correlogram describing the distance of samples and thereby showing their similarity was prepared. Samples were allowed to cluster in an unsupervised way. (C) All upregulated DEGs with an adjusted p-value <0.05 and log2FC >1 were analyzed for over-represented gene ontology (GO) terms (Raudvere et al. 2019). GO terms with a size between 4 and 1000 genes were included in further analysis. The first (lowest p-value) 50 GOs of the category biological process were further assigned to the four key biological processes “neurodevelopment”, “synapse”, “neurotransmitter”, and “neuronal subtype (glutamate)”. In the table all GOs

included in a key biological process are listed with their term name and their corresponding term id. (D) As one gene can be present in different GOs, multiple entries of the same gene in one key biological process are possible. Genes with multientries were counted only once per key biological process. The genes included in the four key biological processes were compared and overlays were performed. (E) The table shows the five genes from the key biological process “neuronal subtype (glutamate)” which are only included in this term and not in one of the other three biological processes, the fold change and a short comment on the respective gene is given.

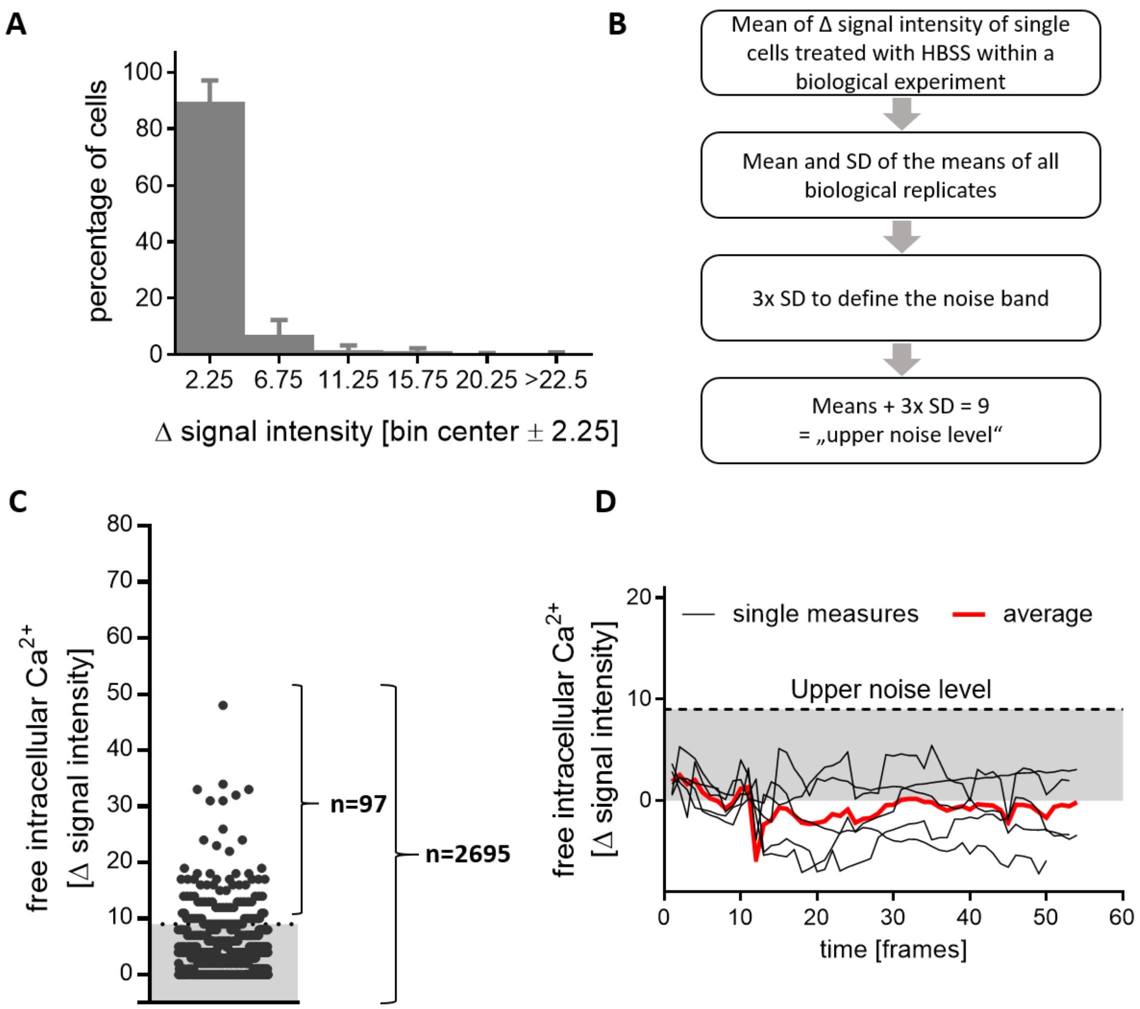

Supplementary figure 4: Determination of reactivity threshold

MCCs were differentiated for 24 days and subsequently used for Ca^{2+} imaging. Stimulation with the negative control Hank's balanced salt solution (HBSS) was performed to determine the background noise and the threshold of reactivity. (A) Changes of Ca^{2+} indicator fluorescence (=delta signal intensity values) of cells treated with HBSS were binned (bin size 4.5), and data are shown as percentage of total cells analyzed. Data are means ± SD; n=6 (B) Workflow to determine the noise band of HBSS and of the threshold of Δ signal intensity to discriminate negative and positive responses. (C) Delta signal intensity of each individual cell treated with HBSS on DoD24. The grey area under the dotted line represents the noise band. All values above the threshold of 9 are counted as reactive cells. Measurements for 2695 individual cells from six biological replicates are shown. False positive were 3.6%. (D) Ca^{2+} signaling traces of five different measurements are shown in black, the red trace represents the average of all single measurements. The grey area under the dotted line represents the noise band.

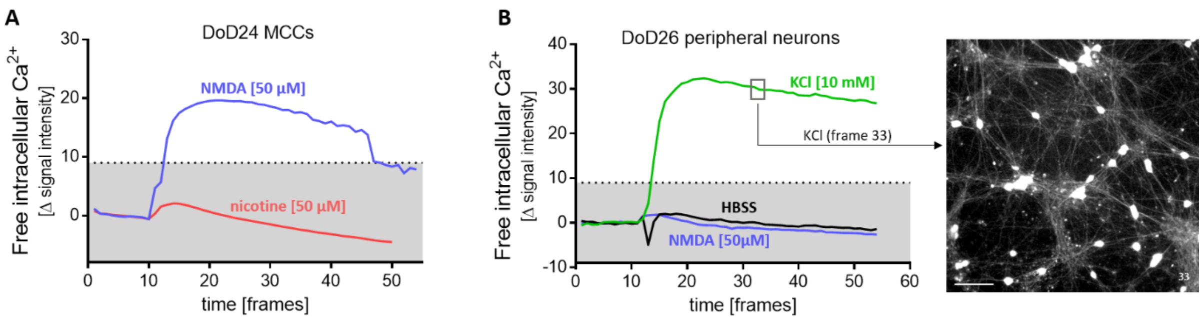

Supplementary figure 5: Specificity controls of Ca^{2+} signaling in MCCs and peripheral neurons

(A) MCCs were differentiated for 24 days and subsequently used for Ca^{2+} imaging. To test for specificity, NMDA [50 µM] or nicotine [50 µM] were administered and free intracellular Ca^{2+} was measured. Traces of the mean Δ signal intensity from >20 cells are shown. (B) Peripheral neurons were differentiated for 26 days according to Hoelting *et al.* (Hoelting et al. 2016) and also used for Ca^{2+} imaging. Changes in free intracellular Ca^{2+} were measured after the administration of KCl [10 mM], NMDA [50 µM] and HBSS. An image of cells after the treatment with KCl [10 mM] illustrates the neuronal phenotype the cells have on DoD26. Data are averages from >50 cells.

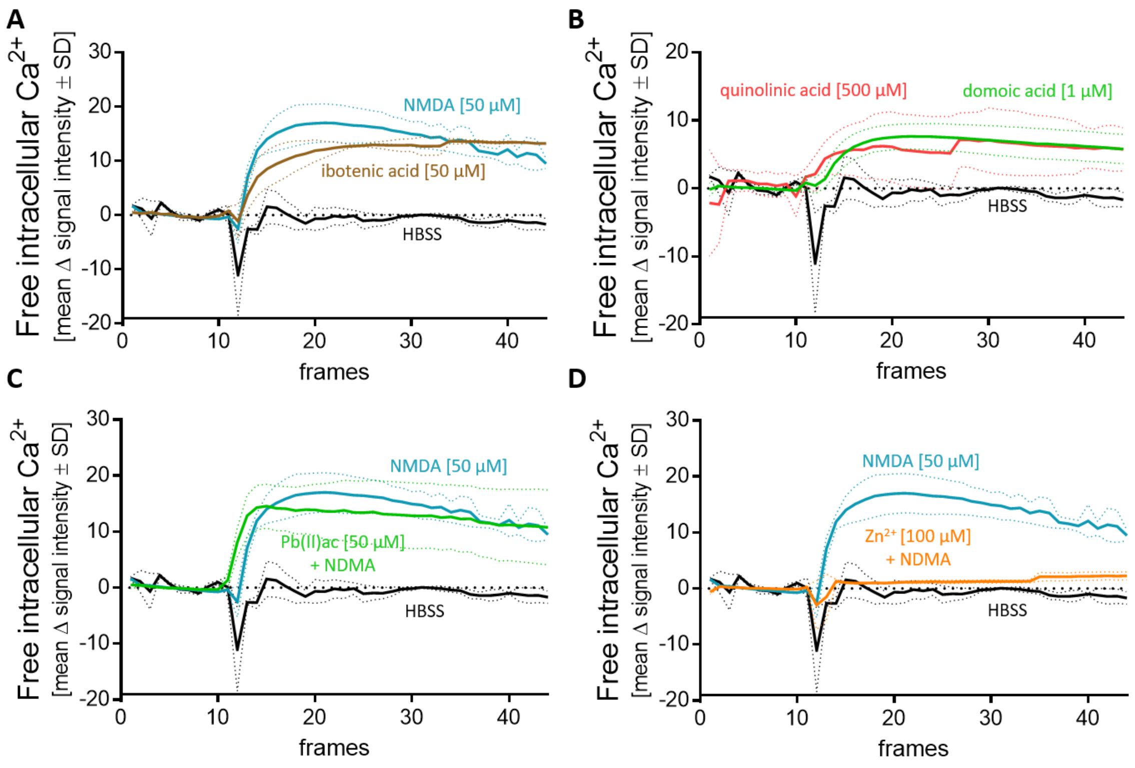

Supplementary figure 6: Ca^{2+} traces of MCCs to characterize their NMDA receptor

MCCs were differentiated until DoD24 and subsequently Ca^{2+} imaging was performed. Changes in free intracellular Ca^{2+} were measured after the administration of the different NMDA receptor agonist: (A) NMDA [50 µM], ibotenic acid [50 µM], (B) quinolinic acid [500 µM], and domoic acid [1 µM]. Cells were pre-incubated for 30 minutes with (C) Pb(II)ac [50 µM] and (D) with Zn^{2+} [100 µM] a known NMDA receptor antagonist and subsequently stimulated with NMDA [50 µM]. Data are averages from 2-4 biological replicates, the dotted lines represent the SD.

Supplementary References

Hoelting L, Klima S, Karreman C, et al. (2016) Stem Cell-Derived Immature Human Dorsal Root Ganglia Neurons to Identify Peripheral Neurotoxicants. Stem cells translational medicine 5(4):476-87 doi:10.5966/sctm.2015-0108

Raudvere U, Kolberg L, Kuzmin I, et al. (2019) g:Profiler: a web server for functional enrichment analysis and conversions of gene lists (2019 update). Nucleic Acids Res 47(W1):W191-w198 doi:10.1093/nar/gkz369

Reinhardt P, Glatza M, Hemmer K, et al. (2013) Derivation and expansion using only small molecules of human neural progenitors for neurodegenerative disease modeling. PLoS One 8(3):e59252 doi:10.1371/journal.pone.0059252
